# Sex differences in trajectories to cardio-renal and mental health outcomes following the onset of type 2 diabetes: An observational cohort study using multi-state analysis in the UK Clinical Practice Research Datalink

**DOI:** 10.1371/journal.pmed.1005196

**Published:** 2026-08-25

**Authors:** Fabiola Eto, Miriam Samuel, Daniel Stow, Rafael Henkin, Alisha Angdembe, Michael R. Barnes, Sarah Finer, Rohini Mathur

**Affiliations:** 1 Wolfson Institute of Population Health, Queen Mary University of London, London, United Kingdom; 2 William Harvey Research Institute, Queen Mary University of London, London, United Kingdom; Wuqu’ Kawoq | Maya Health Alliance, GUATEMALA

## Abstract

**Background:**

Type 2 diabetes (T2D) presents a significant global health challenge, and its prevalence is expected to rise. T2D is associated with diverse complications, including cardiovascular diseases (CVD), end-stage renal disease (ESRD), and mental health disorders like depression and anxiety. Previous research has demonstrated sex differences in the metabolic risk profile, clinical characteristics, and care related to T2D and its complications, including mental health conditions. In this study, we aimed to describe the trajectories of hypertension, cardiovascular diseases, ESRD and mental health conditions in women and men from different age groups with T2D.

**Methods and findings:**

Using individual-level anonymised linked primary and secondary electronic health record (EHR) data from the United Kingdom (UK) Clinical Practice Research Datalink (CPRD) GOLD, we carried out an observational cohort study of 28,720 individuals with incident T2D between 2010 and 2020. We applied competing risk semi-Markov multi-state analysis to investigate and understand the different transitions to cardiovascular disease, ESRD, and mental health conditions following the onset of T2D in women and men. During a median follow-up of 6.8 years, 39% of the cohort moved to another health state. Men constituted 62% of the cohort, with a median age of 54 (IQR 46, 64) at T2D onset, while women were typically older at diagnosis (mean = 56, IQR = 45, 67). Disease trajectories differed significantly by sex: women were more likely than men to be diagnosed with mental health conditions post-T2D, (8.1% versus 5.3%), while men were more likely to be diagnosed with CVD/ESRD (8.4% versus 5.3%) and hypertension (21.1% versus 20.2%) trajectories than women. Women showed higher average of health service utilisation and long-term prescribing than men. Women experienced a later onset of events compared to men. Both women and men who had a combination of T2D and a mental health condition experienced premature mortality compared to other trajectories. Hypertension was the most frequent event following T2D onset across all age groups and sexes. As a study limitation, some of the observed differences in mental health diagnoses between women and men may reflect differences in healthcare-seeking behaviour rather than true differences in the underlying proportions of these conditions.

**Conclusions:**

Our study highlights sex-specific differences in disease trajectories following T2D onset, emphasising the need for stratified healthcare interventions. Women exhibited greater vulnerability to mental health conditions post-T2D, while men showed higher proportion of cardiovascular and renal complications, and had earlier onset of most events compared to women. Both sexes experienced earlier mortality when T2D was combined with a mental health condition. Current UK medical guidelines lack sex-specific prevention strategies and management for T2D and comorbidities, especially mental health conditions. Our findings draw attention to the importance of tailored management strategies that consider sex, age, and the complex interplay of physical and mental health in T2D care.

## Author summary

**Why was this study done?:**

- T2D affects over 4 million people in the UK and is known to increase the risk of heart disease, kidney failure, and mental health conditions, such as depression and anxiety, but previous research has mostly examined these complications in isolation, rather than tracking the order and timing in which they tend to appear overtime. Anticipating the order and timing of comorbidities following T2D represents a valuable window of opportunity for early intervention, and may have meaningful implications for how clinicians respond, even if these population-level patterns will not apply uniformly to every individual.
- Women and men are known to experience T2D differently, yet no large population-based study had previously mapped the full sequence of conditions that develop after a T2D diagnosis separately for each sex and across different age groups.

**What did the researchers do and find?:**

- We followed nearly 29,000 people newly diagnosed with T2D between 2010 and 2020 using routinely collected UK primary and secondary care records, applying a statistical method called multi-state modelling to track the order, timing, and combination of conditions each person developed after their diagnosis of T2D.
- Women were more likely than men to have a mental health condition diagnosed (anxiety, depression, or somatoform disorder) after T2D onset (8.1% versus 5.3%), while men were more likely to receive a diagnosis of cardiovascular disease or kidney failure (8.4% versus 5.3%); hypertension was the most common complication in both sexes across all age groups.
- Both women and men who had a mental health condition after T2D died earlier than those in any other disease trajectory; notably, men in this group died on average 9 years younger than women following the same sequence of conditions, despite using healthcare services considerably less.

**What do these findings mean?:**

- These findings indicate that knowing a complication is likely to occur is not enough; knowing when complications tend to occur and in what order may help translate population-level evidence into more targeted clinical action.
- Routine mental health screening should be incorporated in T2D care practice—particularly for younger women—while earlier cardiovascular and kidney disease monitoring and stronger healthcare engagement strategies are needed for men.
- Our results raise the hypothesis that the combination of T2D and mental health conditions may represent a high-risk group associated with premature death and high healthcare use, pointing to an urgent need for integrated physical and mental healthcare pathways.
- This study has some important limitations to consider: its descriptive design cannot establish causation, requiring future causal studies to confirm whether observed trajectories reflect true causal relationships. We could not examine causes of death separately. Ethnicity could not be analysed due to the small numbers of individuals in each trajectory. We cannot rule out that some of the observed differences in mental health diagnoses between women and men may reflect differences in healthcare-seeking behaviour rather than true differences in the underlying proportions of these conditions.

## Abstract 3

In an observational study, Fabiola Eto and colleagues investigate the trajectories of hypertension, cardiovascular diseases, end-stage renal disease and mental health conditions in women and men from different age groups with T2D in a UK cohort.

## Introduction

Type 2 diabetes is a complex metabolic disorder characterised by high blood glucose, which results from insulin resistance or inadequate insulin production.

Globally, T2D affects approximately 536.6 million individuals (10.5% worldwide) between 20 and 79 years old, a number projected to increase to over 700 million by 2045 [1]. Within the United Kingdom, T2D constitutes an alarming health challenge, with an estimated 4.3 million individuals diagnosed (6.4% of the UK population in 2021), and an estimated additional 850,000 undiagnosed cases (1.3%) [2]. This epidemic imposes substantial economic burdens on healthcare systems and significantly impacts individuals’ quality of life [3].

T2D can lead to a variety of micro and macrovascular conditions, such as kidney and cardiovascular disease, coronary artery disease, stroke, and peripheral vascular disease, which are major causes of illness and death among people with T2D [4–6]. Ultimately, T2D can cause end-stage renal disease (ESRD), requiring renal replacement therapy for many affected individuals [7,8].

There is evidence of a bidirectional relationship between T2D and mental health disorders [9,10]. Depression and anxiety are twice as common among individuals with T2D than in those without it [11,12]. This not only makes managing the disease more challenging but also increases the risk of adverse health outcomes. Similarly, individuals with mental health conditions, especially depression and anxiety, have been found at a nearly 2-fold increased risk for developing T2D [13,14]. Living with a mental health condition can further increase the risk of metabolic dysfunction and poor glycaemic control. A study using data from the Swedish Twin Registry found that major depression was associated with a 32% increased likelihood of T2D among twins aged 40–55, even after considering genetic risk; while T2D was linked to a 33% increased likelihood of major depression among younger twins, but not older ones. Common unique environmental factors were found to contribute to the association between these two conditions [9].

Somatoform disorders are highly relevant in studying mental health trajectories in T2D due to their overlap with common diabetes-related symptoms, impact on healthcare service utilisation, and strong links to psychological distress [15]. Patients with T2D often report unexplained physical symptoms, which may contribute to increased medical consultations and difficulties distinguishing between somatic and diabetes-related symptoms. Evidence suggests shared biological mechanisms, including inflammation, autonomic dysregulation, and neurotransmitter imbalances, which may indicate a bidirectional relationship between metabolic dysfunction and somatisation [16]. Furthermore, somatoform disorders have been linked to an increased risk of depression and anxiety, both of which are associated with poorer diabetes outcomes [17].

Previous research has demonstrated sex differences in the metabolic risk profile, clinical characteristics and care related to T2D and its complications, including mental health conditions [18–21]. This emphasises the need for personalised risk assessment, prevention, and management approaches. However, existing studies have largely focussed on individual comorbidities in isolation, without capturing the ordered sequence in which multiple conditions accumulate over time following a T2D diagnosis. That said, how longitudinal disease trajectories following a diagnosis of T2D differ by sex is poorly understood. Here, we use the term ‘disease trajectory’ to refer to the successive acquisition of health conditions—such as hypertension, mental health conditions, and cardiovascular or renal disease—in the years after T2D onset, including the timing, order, and combination of these events. Additionally, understanding the natural history of these diagnoses—including the timing and sequencing of cardio-renal and mental health conditions—is clinically important, as it can reveal critical windows for intervention and inform how healthcare services should be organised to meet the needs of people living with T2D.

Therefore, following clinical discussions, we carefully selected a set of conditions that are common in the long-term health pathway of individuals with T2D. This study aims to describe the sex-specific trajectories to cardio-renal and mental health outcomes following the onset of T2D to support targeted interventions and personalised healthcare delivery for men and women with T2D across different age groups.

## Methods

### Study design and data sources

We conducted a population-based cohort study using the UK CPRD GOLD [22]. The source population comprised nearly 4 million individuals aged 16 years and over, actively registered with an English general practice (GP) between 2010 and 2020, whose data met CPRD’s acceptable data quality standards. We obtained anonymised historical medical data for the entire source population. The study follow-up period started on January 1, 2010, and continued until December 31, 2020 or the date of leaving the GP practice, last data collection, or death. We obtained linkage to Hospital Episode Statistics Admitted-Patient Care (HES-APC) data, to Office for National Statistics (ONS) mortality data and area-level deprivation data (Index of Multiple Deprivation (IMD)) through CPRD.

This study is reported in accordance with the Reporting of Studies Conducted using Observational Routinely-Collected Data (RECORD) guideline (S1 Checklist). We followed a flexible pre-specified analysis plan (S1 Protocol). This protocol was deliberately broad, designed to support a wider multimorbidity research programme covering ~220 long-term conditions and trajectory analyses across the life course. It explicitly anticipated that, after an initial clustering stage (which has been published in PLOS Medicine [23]), subsequent analyses would focus on a defined subset of conditions selected to ensure we generate new knowledge, allowing flexibility within the bounds of the original protocol. As the project progressed, we narrowed this broad framework to a more specific question: characterising long-term health trajectories following the onset of type 2 diabetes, using multi-state semi-Markov modelling. This reflects a data-driven specification of the protocol’s general trajectory analysis aim, rather than a departure from it.

This study was conducted using data from CPRD, which holds broad ethics approval from the Health Research Authority’s East Midlands – Derby Research Ethics Committee (REC reference 21/EM/0265) covering purely observational research using anonymised primary care data. This approval covers the current study, and study-specific ethics approval was therefore not required. Individual patient consent was not required because CPRD data are provided to researchers in anonymised form, so patients cannot be identified by the research team. The study protocol was additionally reviewed and approved by CPRD’s Independent Scientific Advisory Committee for the Medicines and Healthcare products Regulatory Agency (protocol no. 21_000345).

### Cohort selection and conditions investigated

To identify the trajectories of cardio-renal and mental health conditions following the onset of T2D, we selected all individuals in the source population with a diagnosis of at least one of the following conditions: T2D, hypertension, anxiety, depression, somatoform disorder, atrial fibrillation and flutter, cerebrovascular disease, coronary heart disease, ESRD, heart failure and peripheral arterial disease. We also mention these conditions as “events” throughout this article. We used all data from the individual’s primary and secondary care health records to identify each condition using the first relevant code ever recorded –it could be either a Read code or an ICD-10 (International Classification of Diseases, 10th Revision) code. If a condition was not coded on the electronic health record (EHR), it was considered absent. We conducted a clinical consensus exercise to develop codelists to select the health conditions in the patients’ EHR. Upon clinical discussions, we carefully selected a few diseases that are often found in the long-term health pathway of individuals with T2D and that impact healthcare service utilisation. The codelists used to identify the conditions are available on a public GitHub repository [24].

To compose our cohort, we included only individuals whose index condition (e.g., first condition recorded among the conditions of interest) was type 2 diabetes so we could follow their disease trajectory from this onset. We excluded individuals whose diagnosis was recorded before 2010. This was done to prevent selection bias related to individuals who had died before 2010, as our study only covers mortality data from 2010 to 2020 and includes all individuals actively registered with a GP during the same period.

**Fig 1:**
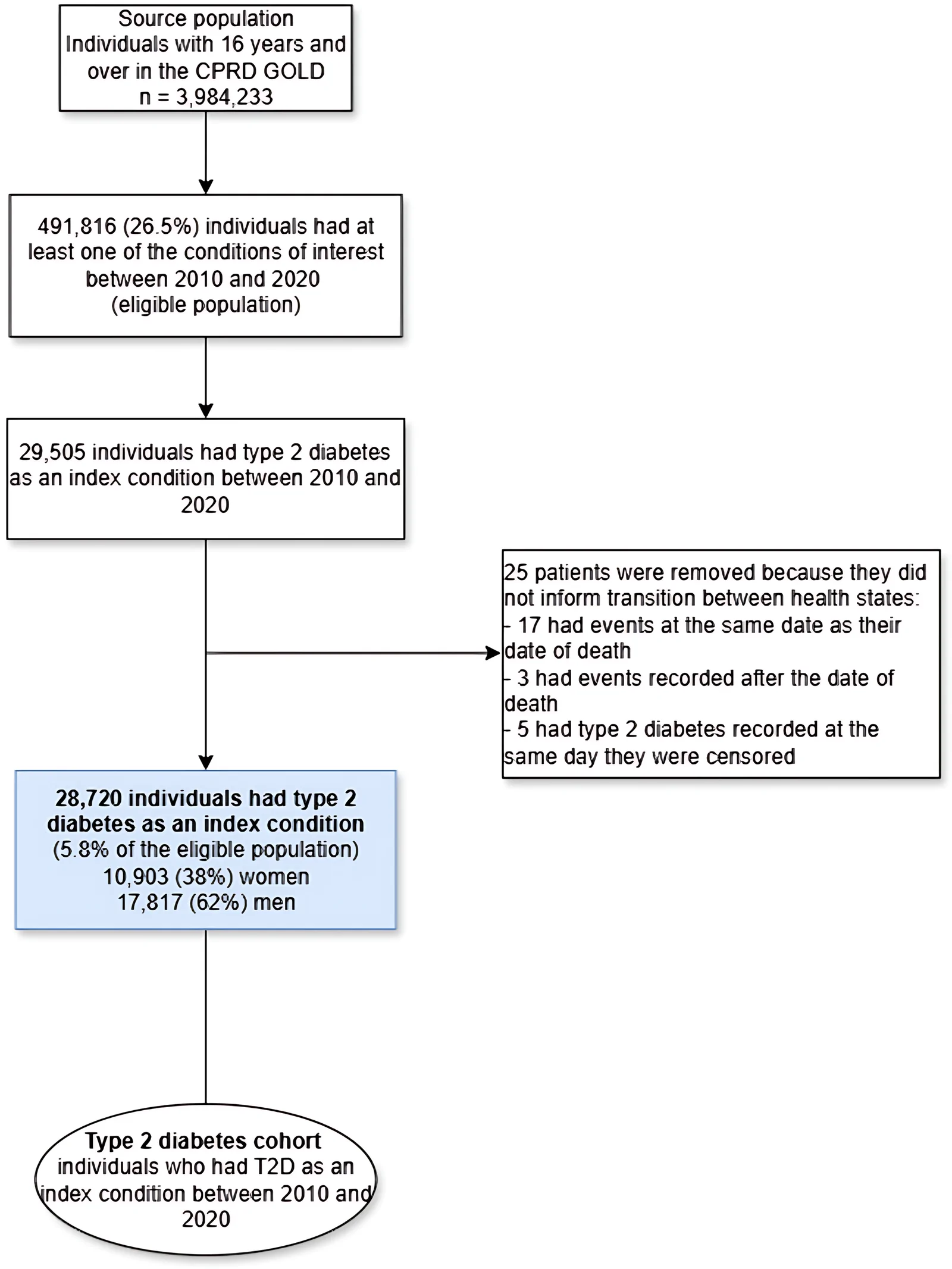
Type 2 diabetes cohort selection flowchart. *The conditions of interest are described in the methods section.

We excluded patients who (i) experienced any of the outcome events of interest prior to T2D diagnosis, (ii) had events recorded for the first time on the same date of their death, (iii) had events recorded after their date of death, and (iv) had the onset of type 2 diabetes on the same day they were censored (Fig 1). These patients were removed because they did not inform transitions between health states.

### Stratification variables

Age at onset of type 2 diabetes and sex were the stratification variables of interest. Individuals were grouped into three age-at-onset categories (16–39, 40–59, and 60 years and above) to allow comparison of disease trajectories across groups with substantially different baseline risk profiles. Age at onset was calculated by subtracting the year of birth from the year of T2D diagnosis, and median age at entry into each subsequent health state was used to describe the timing of disease progression within each trajectory.

### Disease trajectories

Health state occupancy probability –the transition to the next health states following the T2D onset –was the primary outcome of interest in our study. A new health state was ascertained as every new diagnosis recorded for hypertension, ADS (anxiety, depression or somatoform disorder), and CVD/ESRD (atrial fibrillation and flutter, cerebrovascular disease, coronary heart disease, ESRD, heart failure or peripheral arterial disease), and death after the onset of T2D. To simplify the analysis, we grouped the mental health conditions together, and the cardiovascular conditions together with ESRD. All-cause mortality was identified based on the entry of the date of death in the ONS dataset. The transition rates between health states given by the average time in years to the next event were also an outcome of interest in our study.

### Socioeconomic deprivation and health service utilisation

Socioeconomic deprivation was described using the IMD in quintiles, where the first quintile represents the least deprived areas and the fifth quintile, the most deprived. We described health service utilisation through the average number of primary care consultations per individual (defined by the dates of consultation with any primary care clinician and regardless of the type of consultation), and the average number of hospitalisations (defined by the discharge dates related to admitted-patient care), both recorded between 2010 and 2020. Long-term prescribing was defined as the counts of unique prescriptions per British National Formulary (BNF) subparagraphs [25], prescribed 3 or more times per year for the period between 2010 and 2020 [23].

### Statistical analysis

**Fig 2:**
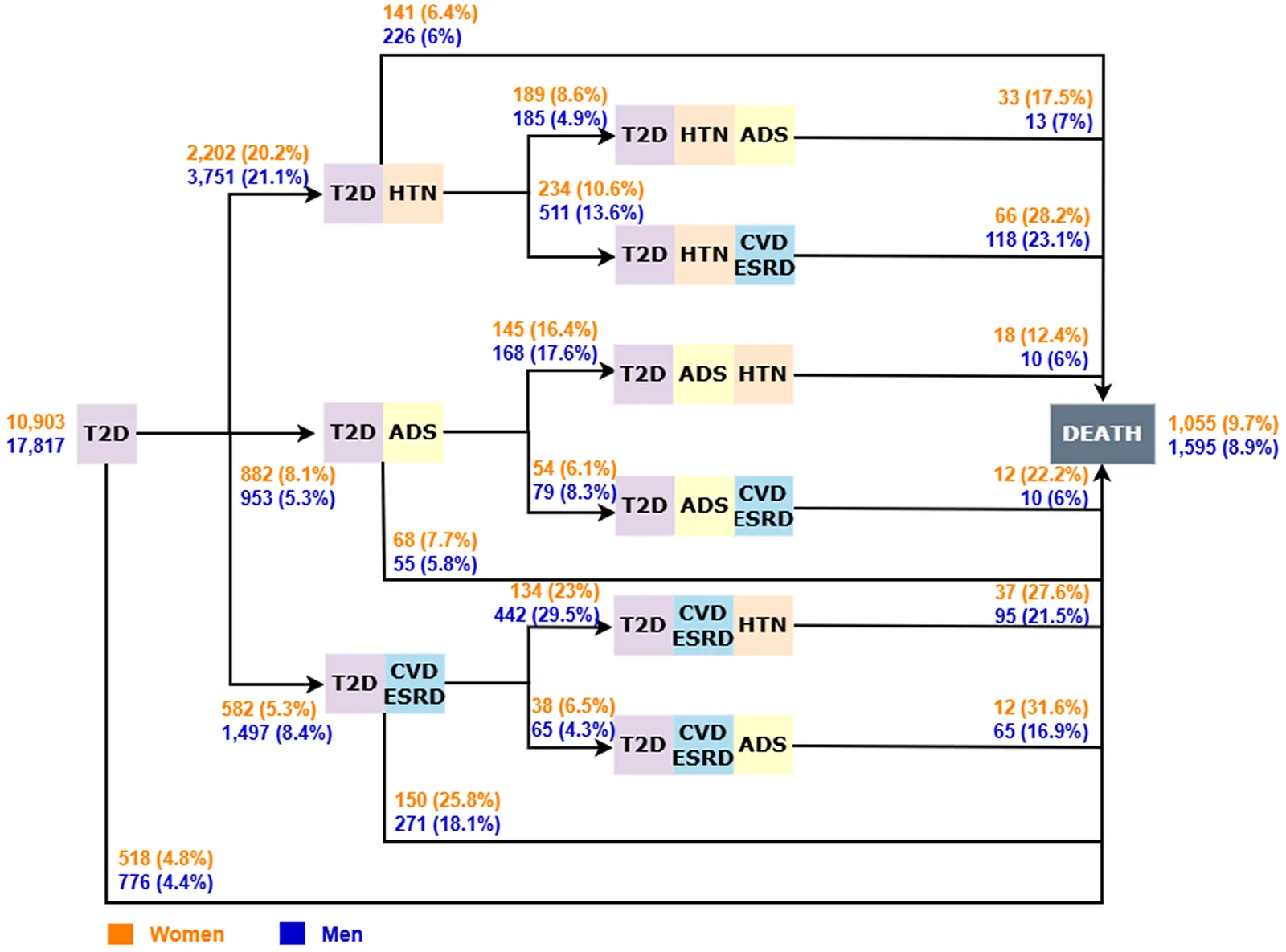
Multi-state diagram: number and proportion of women and men who had each competing next state. * The boxes represent the 11 possible states, and the arrows represent the 19 transitions. ADS, anxiety, depression or somatoform disorder. CVD, atrial fibrillation and flutter, cerebrovascular disease, coronary heart disease, heart failure or peripheral arterial disease. ESRD, end-stage renal disease. HTN, hypertension. T2D = type 2 diabetes.

We used parametric semi-Markov multi-state modelling with Weibull distribution to model the trajectories of mental health, cardiovascular conditions plus ESRD and death after the onset of T2D. Death was the only absorbing state (no transitions allowed after this state) in our model. The multi-state models comprised 11 states and 19 submodels representing competing risks of the next event for each of the 19 transitions following the onset of T2D (Fig 2). The competing risk multi-state model is a special case of survival analysis that represents how an individual moves from a starting state (e.g., type 2 diabetes) to multiple possible states (e.g., hypertension, cardiovascular conditions plus ESRD, mental health conditions, and death) in a continuous time [26].

The Markovian property was assessed using the AUC.test (from the R package *markovMSM*) and did not hold (null hypothesis: the next state to be visited and the time that occurs only depend on the present state and not on any of the previous states visited or the time spent in previous states). Because the Markov assumption did not hold, we adopted semi-Markov (‘clock-reset’) models as a clinically interpretable and parsimonious extension that assumes that the time scale depends on the time spent in the previous state.

All the analyses and data visualisation outputs were done inside Queen Mary University of London’s high-performance computer cluster environment using the R packages *mstate*, *flexsurv*, *ggplot2* and *ggridges* in RStudio (version 2022.02.3 +492 “Prairie Trillium” Release) and R (version 4.2.0). We applied statistical disclosure control to counts describing seven or fewer individuals to avoid any primary or secondary data disclosure using the OpenSafely guidelines [27]. Any counts describing seven or fewer patients were redacted to avoid primary disclosure, and the complimentary values that could be combined with other results to reveal information about individuals or groups were rounded to the nearest five. This approach aligns with the UK Data Protection Act 2018 [28] and the General Data Protection Regulation (GDPR) [29], which emphasise the importance of safeguarding patient confidentiality and ensuring that personal data is not identifiable.

## Results

### Cohort population and characteristics of individuals who had an event following the onset of type 2 diabetes

The CPRD GOLD source population consisted of 3,984,233 individuals aged 16 years and over, actively registered with an English GP between 2010 and 2020. Among these individuals, 29,505 had type 2 diabetes as an index condition. After excluding 25 individuals because they did not inform transition between health states, a cohort of 28,720 individuals was identified (Fig 1).

Sixty-two percentage of the cohort were men, with an average age of 55 at the onset of T2D. Women were, on average, 2 years older than men at T2D onset (56 and 54 years old, respectively) and 4 years, on average, older than men at death (75 *versus* 71 years old). Men and women had a similar distribution of deprivation quintiles, but women were slightly more socioeconomically deprived than men (21% and 19%, respectively, belonged to the most deprived IMD quintile), had more long-term prescribing in a year interval, and had higher average number of consultation in primary care (average of 92 *versus* 32) and hospitalisations (average of 3 *versus* 1) than men in the overall period (2010–2020). The median follow-up time was slightly longer for men than for women (6.9 *versus* 6.6 years), and both had a similar proportion of deaths in the study period (women 10% and men 9%) (Table 1).

**Table 1:** Characterisation of the overall type 2 diabetes population (*n* = 28,720) and those who had an event following the onset of type 2 diabetes (*n* = 11,161) between 2010 and 2020.

	Overall T2D population			Population with events after T2D onset		
**Total**	**Women**	**Men**	**Total**	**Women**	**Men**
**Total**	28,720	10,903 (38)	17,817 (62)	11,161 (39)*	4,184 (37)	6,977 (63)
**Age at onset of T2D**	55 (46, 65)	56 (45, 67)	54 (46, 64)	60 (49, 70)	62 (49, 73)	59 (49, 68)
**Age group at T2D onset**						
16–39	3,923 (14)	1,656 (15)	2,267 (13)	1,017 (9)	435 (10)	582 (8)
40–59	14,314 (50)	4,893 (45)	9,421 (53)	4,871 (44)	1,607 (38)	3,264 (47)
60+	10,483 (36)	4,354 (40)	6,129 (34)	5,273 (47)	2,142 (51)	3,131 (45)
**Age at death**	74 (64, 84)	78 (66, 87)	73 (63, 82)	75 (64, 84)	78 (66, 87)	73 (63, 82)
**IMD (quintiles)**						
1Q (least deprived)	5,413 (19)	1,996 (18)	3,417 (19)	2,065 (19)	768 (18)	1,297 (19)
2Q	5,485 (19)	2,008 (18)	3,477 (20)	2,132 (19)	756 (18)	1,376 (20)
3Q	5,967 (21)	2,302 (21)	3,665 (21)	2,320 (21)	872 (21)	1,448 (21)
4Q	6,155 (21)	2,354 (22)	3,801 (21)	2,439 (22)	934 (22)	1,505 (22)
5Q (greatest deprived)	5,700 (20)	2,243 (21)	3,457 (19)	2,205 (20)	854 (20)	1,351 (19)
**Continuous therapy** ^**1**^	2 (4)	4 (5)	1 (3)	3 (5)	5 (6)	2 (5)
**Consultation**	55 (107)	92 (126)	32 (85)	74 (128)	112 (147)	52 (109)
**Hospitalisation**	2 (7)	3 (8)	1 (7)	3 (10)	4 (9)	2 (11)
**Follow-up length (years)**	6.8 (3.8, 8.9)	6.6 (3.7, 8.9)	6.9 (4, 9)	7.5 (4.6, 9.4)	7.4 (4.4, 9.4)	7.5 (4.8, 9.3)
**Death**	2,650 (9)	1,055 (10)	1,595 (9)	2,650 (24)	1,055 (25)	1,595 (23)

Data are *n* (%) or mean (SD). For age at onset of T2D and at death and follow-up length, data are median (interquartile range).

* Proportion related to the total population

^1^Counts of unique BNF subparagraphs from which an individual had continuous prescribing (3 or more prescriptions that occur in a year).

BNF = British National Formulary. IMD = Index of Multiple Deprivation. Q = quintile. SD = standard deviation. T2D = type 2 diabetes.

During a median follow-up of 6.8 years, 39% (11,161) of the cohort had at least one event after the onset of T2D. Sixty-two% of those were men, slightly older, who had a higher average of consultations and median follow-up time and a slightly higher average number of consultations and use of long-term prescribing compared with the entire T2D cohort. The sex differences remained similar when comparing the entire T2D cohort with those who had an event following the onset of T2D (Table 1).

### Comparing different disease trajectories for women and men after the onset of T2D

Eleven states and 19 unique disease trajectory patterns were identified in our competing risks multi-state models. Fig 2 illustrates these states and trajectories in each sex.

**Fig 3:**
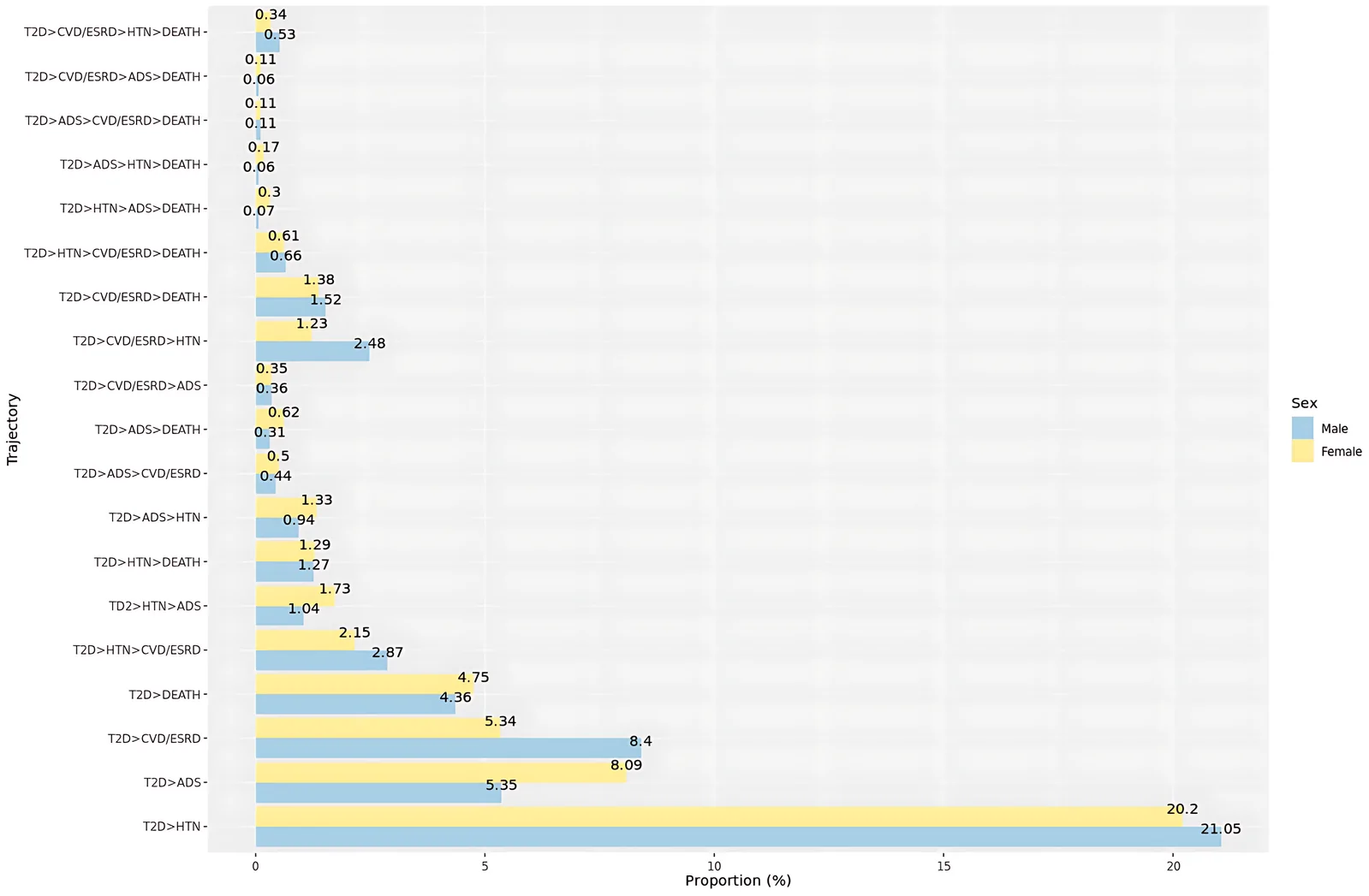
Comparison of different disease trajectories for women and men after the onset of T2D. ADS, anxiety, depression or somatoform disorder. CVD, atrial fibrillation and flutter, cerebrovascular disease, coronary heart disease, heart failure or peripheral arterial disease. ESRD, end-stage renal disease. HTN, hypertension. T2D = type 2 diabetes.

Women had a higher proportion of mental health conditions following the onset of T2D than men (8.1% *versus* 5.3%, respectively). On the other hand, men had a higher proportion of CVD/ESRD (8.4% *versus* 5.3%, respectively) and hypertension (21.1% *versus* 20.2%, respectively) trajectories following the onset of T2D than women (Figs 2 and 3). Both women and men who had hypertension after the onset of T2D were more likely to have a CVD/ESRD diagnosis as the next condition (10.6% and 13.6%, respectively). However, compared to men, women had 1.75 times the probability of having a mental health condition after hypertension (8.6% and 4.9%, respectively). Among women and men who had a mental health condition after T2D onset, men had a higher proportion of individuals developing hypertension (17.6% *versus* 16.4%, respectively) and CVD/ESRD (8.3% *versus* 6.1%, respectively), but women had a higher proportion of mortality by all causes (7.7% *versus* 5.8%).

Among those who had a CVD/ESRD following the T2D onset, women had a higher proportion of mortality by all causes compared to men (25.8% *versus* 18.1%, respectively). A higher proportion of men had a diagnosis for hypertension after that (29.5% *versus* 23%), but women had a higher proportion of mental health conditions than men (6.5% *versus* 4.3%, respectively).

Women had the highest mortality proportions in all trajectories that had death as final outcome, except for the trajectory T2D>ADS>CVD/ESRD, which was higher for men.

Interestingly, in the transitions where ADS is the final health state before death, women had approximately 2-fold increased mortality risk than men. More precisely, 1.87 higher mortality risk in the transition T2D>CVD/ESRD>ADS>DEATH and 2.5 in T2D>HTN>ADS>DEATH (Fig 2).

### Women and men’s sociodemographic characteristics and health service utilisation according to trajectories following the onset of type 2 diabetes

Men and women had similar socioeconomic deprivation distributions across different trajectories. Compared to men, women had a higher average of long-term prescribing in nearly all trajectories; however, their highest average of long-term prescribing occurred in the sequence T2D>ADS > CVD/ESRD (mean = 9, sd= ± 8), and in the trajectory T2D>CVD/ESRD>ADS>DEATH for men (mean = 9, sd= ± 11) (Tables 2–5).

**Table 2:** Women’s age distribution according to trajectories following the onset of type 2 diabetes.

	*n* (%)	Age group at onset			Age at onset of T2D	Age at death
**16–39**	**40–59**	**60+**
**Women’s T2D cohort**	10,903 (100)	1,656 (15)	4,893 (45)	4,354 (40)	56 (15)	75 (14)
**Women who had an event after the T2D onset**	4,184 (38)	435 (10)	1,607 (38)	2,142 (51)	60 (16)	74 (14)
**HTN**	2,202 (20.2)	150 (7)	956 (43)	1,096 (50)	59 (14)	–
HTN > ADS	189 (8.6)	17 (9)	101 (53)	71 (38)	56 (15)	–
HTN > ADS>DEATH	33 (17.5)	0 (0)	9 (27)	24 (73)	70 (13)	76 (13)
HTN > CVD/ESRD	234 (10.6)	[REDACTED]	65 (28)	165 (72)	66 (13)	–
HTN > CVD/ESRD>DEATH	66 (28.2)	[REDACTED]	10 (15)	55 (85)	73 (12)	79 (13)
HTN>DEATH	141 (6.4)	[REDACTED]	30 (21)	110 (79)	72 (14)	77 (14)
**ADS**	882 (8.1)	240 (27)	427 (48)	215 (24)	49 (15)	–
ADS > HTN	145 (16.4)	26 (18)	77 (53)	42 (29)	53 (14)	–
ADS > HTN>DEATH	18 (12.4)	0 (0)	8 (44)	10 (56)	66 (16)	72 (16)
ADS > CVD/ESRD	54 (6.1)	[REDACTED]	20 (25)	30 (75)	60 (17)	–
ADS > CVD/ESRD>DEATH	12 (22.2)	[REDACTED]	[REDACTED]	10 (83)	72 (13)	77 (13)
ADS>DEATH	68 (7.7)	[REDACTED]	20 (29)	50 (71)	67 (15)	71 (15)
**CVD/ESRD**	582 (5.3)	27 (5)	145 (25)	410 (70)	67 (14)	–
CVD/ESRD>HTN	134 (23.0)	[REDACTED]	40 (31)	90 (69)	65 (14)	–
CVD/ESRD>HTN>DEATH	37 (27.6)	[REDACTED]	[REDACTED]	30 (100)	73 (13)	78 (13)
CVD/ESRD>ADS	38 (6.5)	[REDACTED]	10 (29)	25 (71)	64 (16)	–
CVD/ESRD>ADS>DEATH	12 (31.6)	[REDACTED]	[REDACTED]	10 (100)	74 (14)	78 (13)
CVD/ESRD>DEATH	150 (25.8)	[REDACTED]	15 (10)	135 (90)	75 (12)	79 (12)
**DEATH**	518 (4.8)	18 (3)	79 (15)	421 (81)	71 (15)	74 (15)

Data are *n* (%) or mean (SD).

The proportions are related to the total female T2D cohort (*n* = 10,903).

Any counts describing ≤7 patients were redacted to avoid primary disclosure, and the complementary values that could be combined with other results to reveal information about individuals or groups were rounded to the nearest five. This is the reason that for some categorical variables, the counts will not sum up to the total column. The proportions related to the rounded values will sum up to 100%.

ADS = anxiety, depression or somatoform disorder. CVD = atrial fibrillation and flutter, cerebrovascular disease, coronary heart disease, heart failure or peripheral arterial disease. ESRD = end-stage renal disease. HTN = hypertension. T2D = type 2 diabetes.

**Table 3:** Women’s socioeconomic characteristics and health service utilisation according to trajectories following the onset of type 2 diabetes.

	*n* (%)	IMD (quintiles)				5Q (greatest deprived)	Long-term prescribing^1^	Consultation	Hospitalisation
**1Q (least deprived)**	**2Q**	**3Q**	**4Q**
**Women’s T2D cohort**	10,903 (100)	1,996 (18)	2,008 (18)	2,302 (21)	2,354 (22)	2,243 (21)	4 (5)	92 (126)	3 (8)
**Women who had an event after the T2D onset**	4,184 (38)	768 (18)	756 (18)	872 (21)	934 (22)	854 (20)	5 (6)	112 (147)	4 (9)
**HTN**	2,202 (20.2)	415 (19)	396 (18)	438 (20)	508 (23)	445 (20)	5 (6)	114 (148)	4 (8)
HTN > ADS	189 (8.6)	30 (16)	25 (13)	39 (21)	43 (23)	52 (28)	7 (8)	152 (179)	5 (10)
HTN > ADS>DEATH	33 (17.5)	[redacted]	[redacted]	10 (100)	[redacted]	[redacted]	7 (7)	135 (157)	6 (10)
HTN > CVD/ESRD	234 (10.6)	44 (19)	47 (20)	42 (18)	55 (24)	46 (20)	7 (7)	164 (185)	7 (10)
HTN > CVD/ESRD>DEATH	66 (28.2)	14 (21)	13 (20)	14 (21)	12 (18)	13 (20)	8 (9)	178 (218)	8 (10)
HTN>DEATH	141 (6.4)	26 (18)	33 (23)	30 (21)	32 (23)	20 (14)	5 (6)	95 (122)	7 (14)
**ADS**	882 (8.1)	130 (15)	143 (16)	183 (21)	212 (24)	214 (24)	6 (6)	131 (151)	5 (10)
ADS > HTN	145 (16.4)	24 (17)	19 (13)	33 (23)	33 (23)	36 (25)	8 (7)	161 (162)	6 (8)
ADS > HTN>DEATH	18 (12.4)	[redacted]	[redacted]	[redacted]	[redacted]	[redacted]	8 (9)	150 (173)	11 (12)
ADS > CVD/ESRD	54 (6.1)	11 (20)	9 (17)	13 (24)	11 (20)	10 (19)	9 (8)	192 (192)	7 (17)
ADS > CVD/ESRD>DEATH	12 (22.2)	[redacted]	[redacted]	[redacted]	[redacted]	[redacted]	8 (9)	163 (214)	4 (5)
ADS>DEATH	68 (7.7)	12 (18)	15 (22)	14 (21)	13 (19)	14 (21)	5 (6)	96 (119)	10 (23)
**CVD/ESRD**	582 (5.3)	107 (18)	116 (20)	133 (23)	112 (19)	114 (20)	5 (7)	117 (166)	5 (8)
CVD/ESRD>HTN	134 (23.0)	29 (22)	28 (21)	29 (22)	23 (17)	25 (19)	5 (7)	127 (168)	5 (7)
CVD/ESRD>HTN>DEATH	37 (27.6)	8 (22)	10 (22)	10 (24)	[redacted]	10 (22)	5 (7)	82 (115)	7 (10)
CVD/ESRD>ADS	38 (6.5)	[redacted]	10 (33)	[redacted]	10 (33)	10 (33)	7 (8)	147 (182)	9 (11)
CVD/ESRD>ADS>DEATH	12 (31.6)	[redacted]	[redacted]	[redacted]	[redacted]	[redacted]	6 (8)	82 (138)	8 (12)
CVD/ESRD>DEATH	150 (25.8)	22 (15)	33 (22)	43 (29)	23 (15)	29 (19)	4 (7)	93 (145)	4 (10)
**DEATH**	518 (4.8)	116 (22)	101 (19)	118 (23)	102 (20)	81 (16)	3 (5)	66 (102)	5 (12)

Data are *n* (%) or mean (SD).

The proportions are related to the total female T2D cohort (i = 10,903).

^1^Counts of unique BNF subparagraphs from which an individual had continuous prescribing (3 or more prescriptions that occur in a year).

Any counts describing ≤7 patients were redacted to avoid primary disclosure, and the complementary values that could be combined with other results to reveal information about individuals or groups were rounded to the nearest five. This is the reason that for some categorical variables, the counts will not sum up to the total column. The proportions related to the rounded values will sum up to 100%.

ADS = anxiety, depression or somatoform disorder. BNF = British National Formulary. CVD = atrial fibrillation and flutter, cerebrovascular disease, coronary heart disease, heart failure or peripheral arterial disease. ESRD = end-stage renal disease. HTN = hypertension. IMD = Index of Multiple Deprivation. Q = quintile. T2D = type 2 diabetes.

**Table 4:** Men’s age distribution according to trajectories following the onset of type 2 diabetes.

	*n* (%)	Age group at onset			Age at onset of T2D	Age at death
**16–39**	**40–59**	**60+**
**Men’s T2D cohort**	17,817 (100)	2,267 (13)	9,421 (53)	6,129 (34)	54 (13)	71 (13)
**Men who had an event after the T2D onset**	6,977 (39)	582 (8)	3,264 (47)	3,131 (45)	58 (13)	70 (13)
**HTN**	3,751 (21.1)	306 (8)	1913 (51)	1,532 (41)	56 (12)	–
HTN > ADS	185 (4.9)	24 (13)	105 (57)	56 (30)	53 (11)	–
HTN > ADS>DEATH	13 (7.0)	[redacted]	[redacted]	10 (100)	61 (13)	66 (13)
HTN > CVD/ESRD	511 (13.6)	27 (5)	199 (39)	285 (56)	61 (13)	–
HTN > CVD/ESRD>DEATH	118 (23.1)	[redacted]	25 (22)	90 (78)	69 (12)	75 (12)
HTN>DEATH	226 (6.0)	[redacted]	55 (25)	165 (75)	65 (11)	70 (11)
**ADS**	953 (5.3)	199 (21)	576 (60)	178 (19)	49 (12)	–
ADS > HTN	168 (17.6)	25 (15)	108 (64)	35 (21)	51 (11)	–
ADS > HTN>DEATH	10 (6.0)	[redacted]	[redacted]	10 (100)	70 (9)	76 (10)
ADS > CVD/ESRD	79 (8.3)	[redacted]	45 (64)	25 (36)	55 (12)	–
ADS > CVD/ESRD>DEATH	20 (25.3)	[redacted]	[redacted]	15 (100)	63 (14)	68 (14)
ADS>DEATH	55 (5.8)	[redacted]	25 (50)	25 (50)	59 (13)	63 (14)
**CVD/ESRD**	1,497 (8.4)	57 (4)	578 (39)	862 (58)	62 (13)	–
CVD/ESRD>HTN	442 (29.5)	11 (2)	172 (39)	259 (59)	62 (12)	–
CVD/ESRD>HTN>DEATH	95 (21.5)	0 (0)	15 (16)	80 (84)	72 (10)	77 (10)
CVD/ESRD>ADS	65 (4.3)	[redacted]	35 (54)	30 (46)	59 (14)	–
CVD/ESRD>ADS>DEATH	11 (16.9)	[redacted]	[redacted]	10 (100)	66 (18)	71 (18)
CVD/ESRD>DEATH	271 (18.1)	[redacted]	45 (17)	220 (83)	71 (12)	75 (12)
**DEATH**	776 (4.4)	20 (3)	197 (25)	559 (72)	67 (14)	70 (14)

Data are *n* (%) or mean (SD).

The proportions are related to the total male T2D cohort (*n* = 17,817).

Any counts describing ≤7 patients were redacted to avoid primary disclosure, and the complementary values that could be combined with other results to reveal information about individuals or groups were rounded to the nearest five. This is the reason that for some categorical variables, the counts will not sum up to the total column. The proportions related to the rounded values will sum up to 100%.

ADS = anxiety, depression or somatoform disorder. CVD = atrial fibrillation and flutter, cerebrovascular disease, coronary heart disease, heart failure or peripheral arterial disease. ESRD = end-stage renal disease. HTN = hypertension. T2D = type 2 diabetes.

**Table 5:** Men’s socioeconomic characteristics and health service utilisation according to trajectories following the onset of type 2 diabetes.

	*n* (%)	IMD (quintiles)				5Q (greatest deprived)	Long-term prescribing^1^	Consultation	Hospitalisation
**1Q (least deprived)**	**2Q**	**3Q**	**4Q**
**Men’s T2D cohort**	17,817 (100)	3,417 (19)	3,477 (20)	3,665 (21)	3,801 (21)	3,457 (19)	1 (3)	32 (85)	1 (7)
**Men who had an event after the T2D onset**	6,977 (39)	1,297 (19)	1,376 (20)	1,448 (21)	1,505 (22)	1,351 (19)	2 (5)	52 (109)	2 (11)
**HTN**	3,751 (21.1)	718 (19)	733 (20)	792 (21)	800 (21)	708 (19)	2 (5)	52 (109)	2 (6)
HTN > ADS	185 (4.9)	33 (18)	23 (12)	47 (25)	36 (19)	46 (25)	4 (7)	92 (156)	3 (7)
HTN > ADS>DEATH	13 (7.0)	[redacted]	0 (0)	[redacted]	[redacted]	[redacted]	3 (4)	47 (63)	8 (12)
HTN > CVD/ESRD	511 (13.6)	88 (17)	114 (22)	106 (21)	110 (22)	93 (18)	4 (6)	92 (142)	4 (9)
HTN > CVD/ESRD>DEATH	118 (23.1)	16 (14)	32 (27)	27 (23)	21 (18)	22 (19)	5 (7)	93 (138)	6 (13)
HTN>DEATH	226 (6.0)	46 (20)	52 (23)	50 (22)	37 (16)	41 (18)	2 (5)	50 (98)	4 (10)
**ADS**	953 (5.3)	146 (15)	175 (18)	200 (21)	212 (22)	220 (23)	2 (4)	54 (115)	3 (24)
ADS > HTN	168 (17.6)	25 (15)	24 (14)	36 (21)	49 (29)	34 (20)	4 (6)	96 (160)	7 (55)
ADS > HTN>DEATH	10 (6.0)	0 (0)	0 (0)	[redacted]	[redacted]	[redacted]	4 (5)	114 (156)	8 (17)
ADS > CVD/ESRD	79 (8.3)	11 (14)	13 (16)	20 (25)	14 (18)	21 (27)	5 (7)	99 (132)	4 (6)
ADS > CVD/ESRD>DEATH	20 (25.3)	[redacted]	[redacted]	[redacted]	[redacted]	[redacted]	4 (8)	64 (144)	4 (7)
ADS>DEATH	55 (5.8)	[redacted]	15 (30)	10 (20)	10 (20)	15 (30)	3 (5)	60 (121)	3 (6)
**CVD/ESRD**	1,497 (8.4)	277 (19)	310 (21)	307 (21)	320 (21)	283 (19)	3 (5)	63 (121)	3 (8)
CVD/ESRD>HTN	442 (29.5)	88 (20)	81 (18)	101 (23)	86 (19)	86 (19)	4 (6)	88 (140)	4 (9)
CVD/ESRD>HTN>DEATH	95 (21.5)	15 (16)	19 (20)	24 (25)	22 (23)	15 (16)	5 (7)	102 (154)	6 (11)
CVD/ESRD>ADS	65 (4.3)	10 (15)	19 (29)	9 (14)	14 (22)	13 (20)	6 (8)	121 (188)	5 (8)
CVD/ESRD>ADS>DEATH	11 (16.9)	[redacted]	[redacted]	[redacted]	[redacted]	[redacted]	9 (11)	184 (213)	11 (11)
CVD/ESRD>DEATH	271 (18.1)	42 (15)	59 (22)	49 (18)	61 (23)	60 (22)	3 (5)	55 (112)	4 (12)
**DEATH**	776 (4.4)	156 (20)	158 (20)	149 (19)	173 (22)	140 (18)	1 (4)	27 (72)	2 (8)

Data are *n* (%) or mean (SD).

The proportions are related to the total male T2D cohort (*n* = 17,817).

^1^Counts of unique BNF subparagraphs from which an individual had continuous prescribing (3 or more prescriptions that occur in a year).

Any counts describing ≤7 patients were redacted to avoid primary disclosure, and the complementary values that could be combined with other results to reveal information about individuals or groups were rounded to the nearest five. This is the reason that for some categorical variables, the counts will not sum up to the total column. The proportions related to the rounded values will sum up to 100%.

ADS = anxiety, depression or somatoform disorder. BNF = British National Formulary. CVD = atrial fibrillation and flutter, cerebrovascular disease, coronary heart disease, heart failure or peripheral arterial disease. ESRD = end-stage renal disease. HTN = hypertension. IMD = Index of Multiple Deprivation. Q = quintile. T2D = type 2 diabetes.

Women had a much higher average number of primary care consultations in all trajectories over the study period than men, except for the sequence T2D>CVD/ESRD>ADS>DEATH, in which men had a 2-fold higher average of primary care consultations than women (mean = 184, sd= ± 213 *versus* mean = 82, sd= ± 138, respectively).

The average number of hospitalisations over the study period was higher for men compared to women who had the following trajectories: T2D>HTN > ADS>DEATH (8, ± 12 and 6, ± 10, respectively), T2D>ADS > HTN (7, ± 55 and 6, ± 8, respectively), and T2D>CVD/ESRD>ADS>DEATH (11, ± 11 and 8, ± 12, respectively). For all the remaining trajectories, women had a higher hospitalisation average than men over the study period.

### Estimated probabilities and times to next events after the T2D onset according to sex and age groups

**Fig 4:**
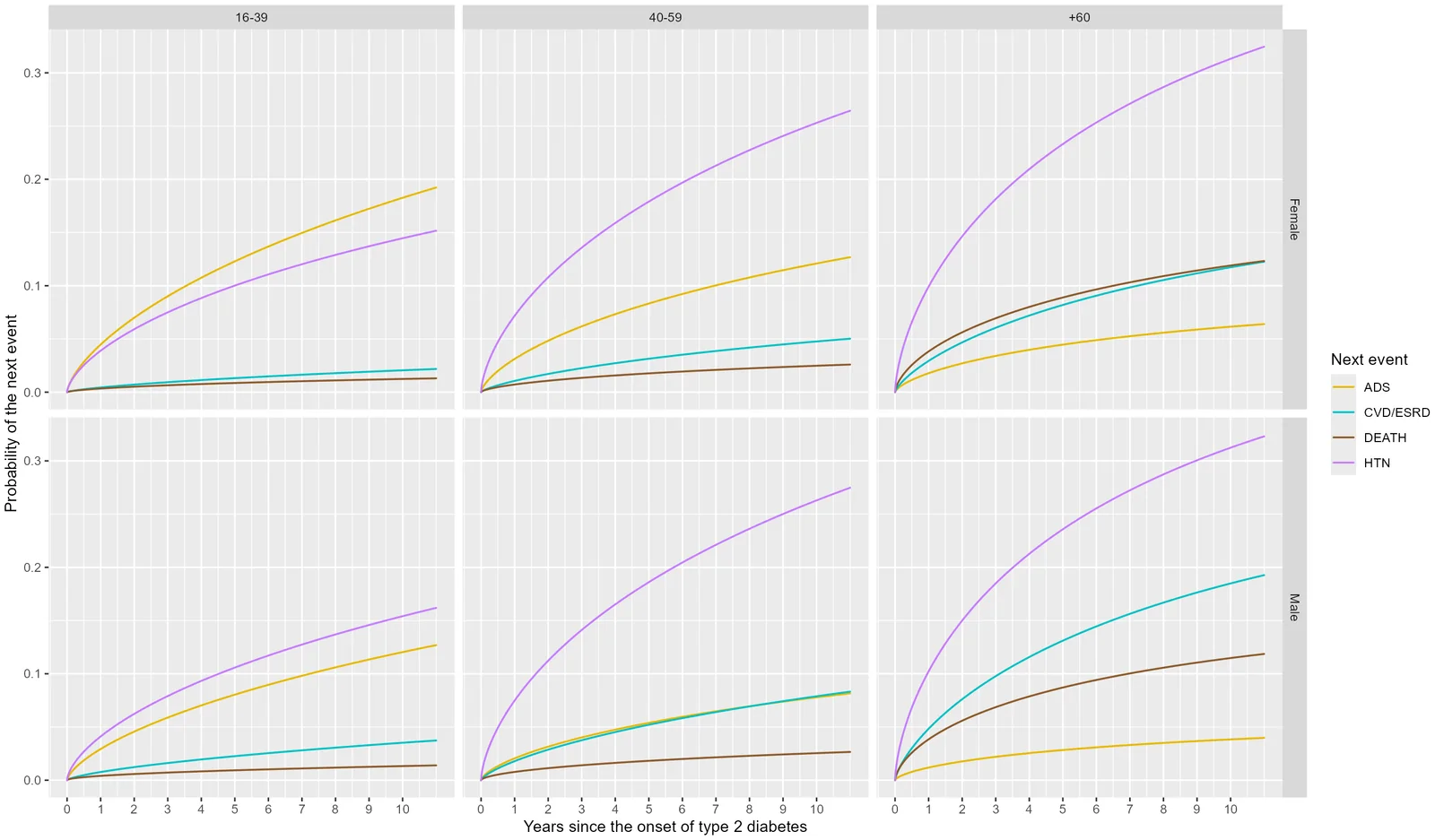
Probabilities of having experienced each competing next state following the onset of T2D against time since the onset of T2D by sex and age groups. ADS = anxiety, depression or somatoform disorder. CVD = atrial fibrillation and flutter, cerebrovascular disease, coronary heart disease, heart failure or peripheral arterial disease. ESRD = end-stage renal disease. HTN = hypertension. T2D = type 2 diabetes.

Women between 16 and 39 years old had the highest probability of having anxiety, depression or somatoform disorder at any time point after the onset of T2D compared to all other age groups. Over time, this probability decreased for women in older age groups, who presented an increasing probability of having hypertension after the T2D onset over the study period. Compared to women aged 16–59 years old, women above 60 had a higher and increasing probability over time of dying or having a CVD/ESRD (Fig 4).

After the onset of T2D, men were more likely to develop hypertension than any other competing event, and this likelihood increased over time with age. Younger men had a higher probability of experiencing a mental health condition than older men. As men aged, their probability of developing CVD/ESRD increased over time. Men over the age of 60 had a higher and increasing proportion of mortality over time compared to men under 60. Men between 40 and 59 years old had a very similar probability of having a mental health condition or a CVD/ESRD over the study period.

After the onset of T2D, women of any age were more likely to be diagnosed with a mental health condition over time in comparison to men in the same age group. However, women had a lower probability of developing CVD/ESRD in any age group when compared to men in the same age group.

For women aged 60 years and above, the probability of developing CVD/ESRD after T2D onset was similar to the probability of death over time. However, the probability of death was slightly higher most of the time. On the other hand, for men, the probability of developing CVD/ESRD after the onset of T2D was higher than mortality over time, and the highest probability among all groups.

The hypertension probability over the study period was very similar among women and men in all age groups.

### Estimated probabilities and times to third events after the second event according to sex and age groups

**Fig 5:**
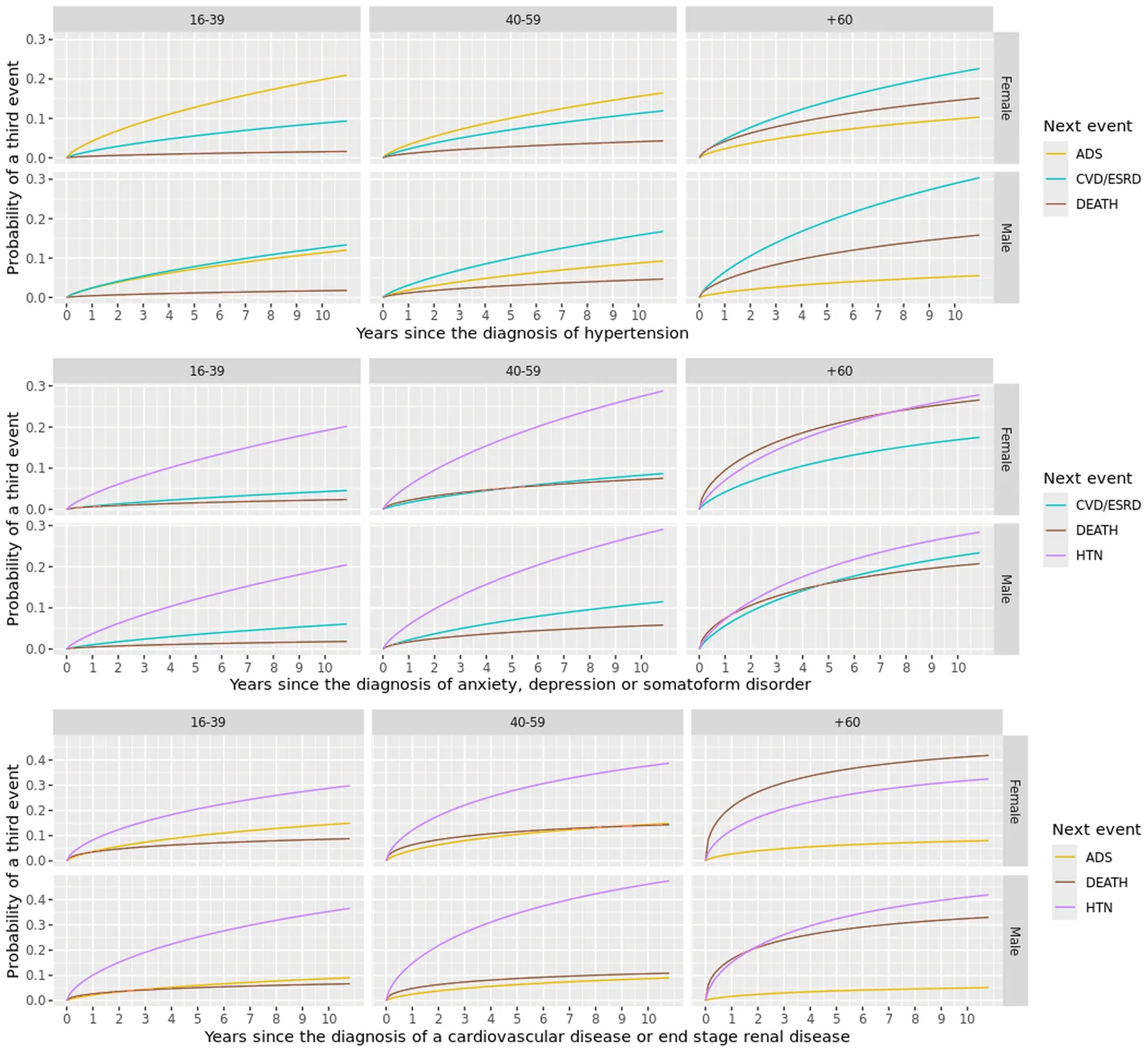
Probabilities of having experienced each competing third state following the second state against time since the diagnosis of the second event by sex and age groups. ADS = anxiety, depression or somatoform disorder. CVD = atrial fibrillation and flutter, cerebrovascular disease, coronary heart disease, heart failure or peripheral arterial disease. ESRD = end-stage renal disease. HTN = hypertension. T2D = type 2 diabetes.

We found that women aged between 16 and 59 years old were more likely to be diagnosed with a mental health condition over time following the diagnosis of hypertension compared to women of other ages and men of the same age group. For women above this age group, the third most likely event was CVD/ESRD. On the other hand, for men, regardless of age group, the most likely event after hypertension was CVD/ESRD, with an increased probability for the oldest age group (Fig 5).

For both women and men below 60 years old who had a mental health condition as the first condition after the onset of T2D, the probability of having hypertension is much higher compared to other competing events. However, for women aged 60 years and older, in the first 6 years after having ADS, the probability of death is higher than any other event. For men over 60, hypertension and death probabilities were similar until the second year after having a mental health condition, when hypertension became the most likely event over the following years.

After a diagnosis of CVD/ESRD following T2D, hypertension was the most likely event over time for both men and women between 16 and 59 years old. However, for women above 60 years old who had a CVD/ESRD, death was the most likely event at any time point over the study period. For men over the age of 60, the probability of death and hypertension were similar until the second year following a CVD/ESRD diagnosis, at which point the likelihood of hypertension increased.

### Median age entering each health state according to trajectory

**Fig 6:**
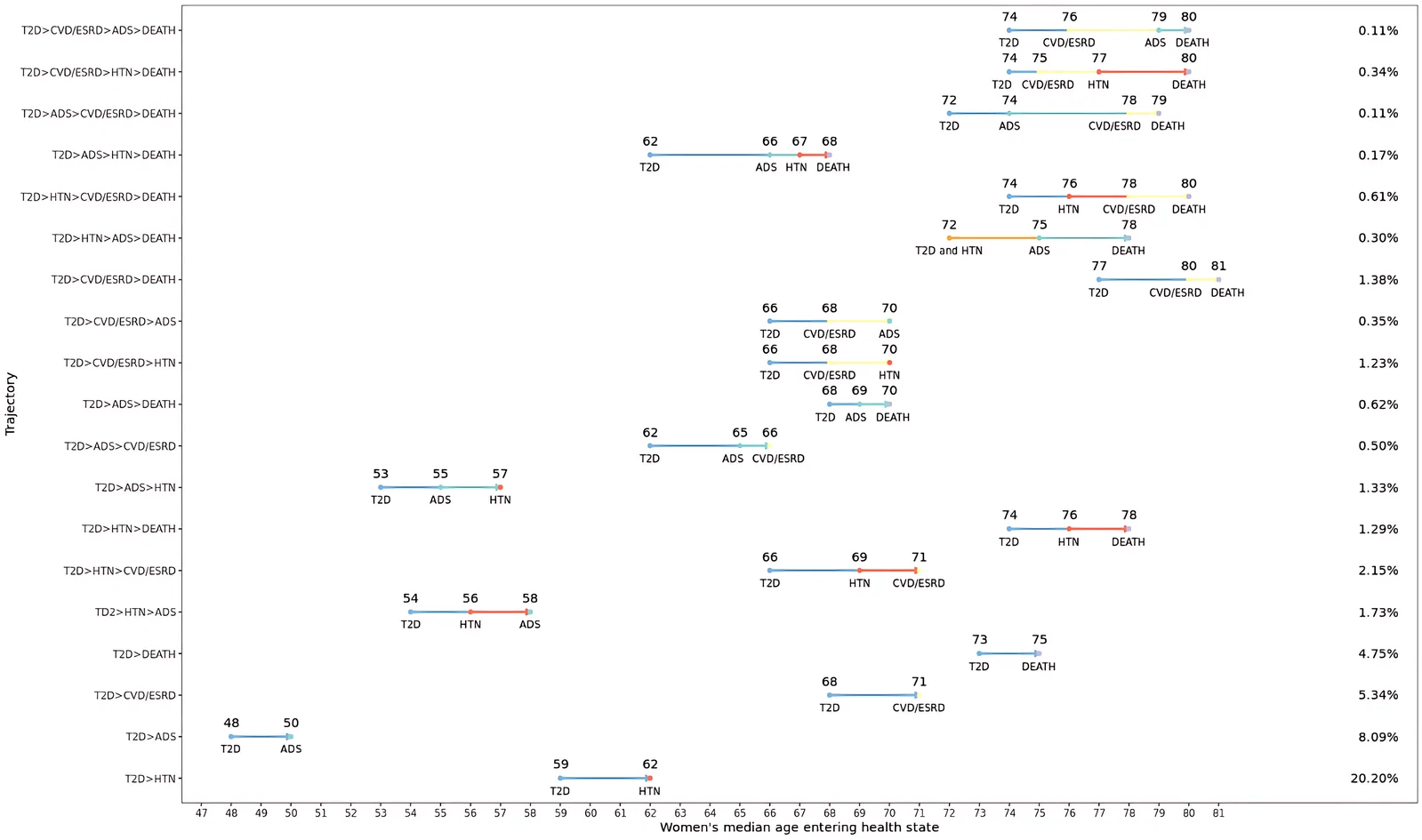
Women’s median age entering each health state. The plot displays trajectories on the left and the respective proportion of women in each trajectory on the right. ADS = anxiety, depression or somatoform disorder. CVD = atrial fibrillation and flutter, cerebrovascular disease, coronary heart disease, heart failure or peripheral arterial disease. ESRD = end-stage renal disease. HTN = hypertension. T2D = type 2 diabetes.

**Fig 7:**
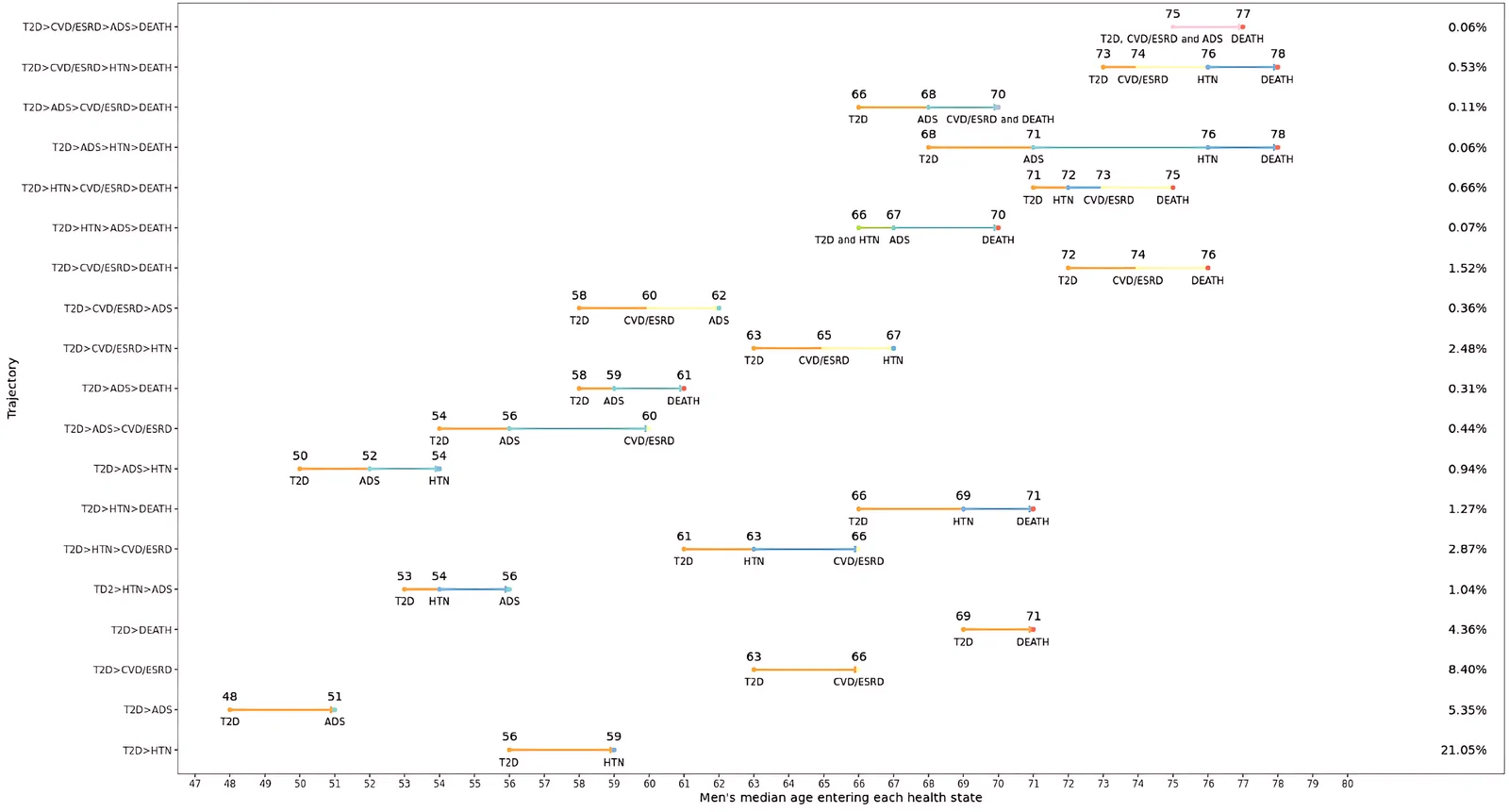
Men’s median age entering each health state. The plot displays trajectories on the left and the respective proportion of men in each trajectory on the right. ADS = anxiety, depression or somatoform disorder. CVD = atrial fibrillation and flutter, cerebrovascular disease, coronary heart disease, heart failure or peripheral arterial disease. ESRD = end-stage renal disease. HTN = hypertension. T2D = type 2 diabetes.

In general, women had a later onset of events after the diagnosis of T2D than men. The median time between health states ranged from 1 to 4 years for women and 1–5 years for men (Figs 6 and 7).

The trajectory T2D>HTN covered 20 and 21% of the women’s and men’s cohort, respectively. Women following this trajectory were likely to progress to T2D at a median age of 59 and develop hypertension at a median age of 62, while men in the same trajectory progressed to T2D at a median age of 56, and developed hypertension at a median age of 59.

The onset of anxiety, depression, or somatoform disorders occurred at the earliest age for both women and men (50 and 51 years old, respectively). Women’s and men’s median age at death was earliest in those with T2D onset followed by a mental health condition (61 and 70 years old, respectively), with men dying 9 years earlier than women following the same trajectory (T2D>ADS>DEATH). Interestingly, for this same trajectory, women had higher average of long-term prescribing, consultation, and hospitalisation (median = 5, 96, and 10, respectively) in a 10-year period compared to men (median = 3, 60, and 3, respectively).

Men had an earlier onset of CVD/ESRD following T2D onset compared to women—men’s earliest median age for having a CVD/ESRD was 60 years old, while women’s was 66 years old.

The longest gap between health states for women was found in the trajectories T2D>ADS>HTN> DEATH and T2D>ADS>CVD/ESRD>DEATH, where there was a median of 4 years between the onset of T2D and ADS and between ADS and a CVD/ESRD. For men, a 5-year gap was found between ADS and hypertension in the trajectory T2D>ADS>HTN>DEATH.

Additional analyses showing the total frequency and proportion of each health event by sex, and analyses for the entire cohort of T2D can be seen in the Supporting Information (S1–S9 Tables and S1–S7 Figs).

## Discussion

In a cohort of nearly 29,000 individuals diagnosed with type 2 diabetes between 2010 and 2020, 39% of them experienced at least one significant health event following T2D onset over a median of 6.8 years of follow-up. We found that trajectory type, timing, and healthcare utilisation all differed by sex. Women were more likely to develop mental health conditions post-T2D and to experience trajectories culminating in death, whereas men showed higher proportions of cardiovascular disease, ESRD, and hypertension. In terms of timing, women generally had a later onset of subsequent health events; mental health conditions occurred earliest in both sexes, CVD/ESRD appeared earlier in men, and both sexes reached their earliest age at death when a mental health condition followed T2D onset. Despite similar socioeconomic circumstances as measured by IMD, women generally presented higher health service utilisation and average of long-term prescribing. Taken together, these findings highlight a pattern in which women accumulate mental health burden earlier while men face earlier cardio-renal deterioration, suggesting the need for tailored interventions in managing T2D-related comorbidities.

We highlighted important differences in disease trajectories between sexes when accounting for disease competing risks. Women of any age group were more likely than men to be diagnosed with a mental health condition after the onset of T2D—with women aged 16–39 years old having the highest probability. In comparison, across all the age groups, men had a higher probability of being diagnosed with a CVD/ESRD than women. Previous studies [30–32] have found that women with T2D present higher proportion of mental health conditions than men with T2D, particularly in disorders such as depression and anxiety, suggesting a gender-specific vulnerability. A Spanish population-level study [30] found that 20.7% of women with T2D experienced depression, compared to 7.57% of men. The complex interplay of biologic, cultural, and diagnostic may partly explain these differences. Women are more likely to have internalising disorders (e.g., anxiety and depression) than men [33]; and social expectations and gender roles can influence the expression of a mental health condition –women may be more likely to report symptoms, while men are less likely to report due to social stigma [33,34].

Our study focussed on the development of health conditions after the onset of T2D. However, it is important to consider that depression is also associated with an increased risk of developing T2D, indicating a bidirectional relationship between depression and T2D [35].

Our observation that men had a higher probability of developing CVD/ESRD in any age group compared to women in the same age group is supported by a few reports. A UK study investigating sex-specific risks for cardiovascular disease across the glycaemic spectrum found that the absolute CVD rates were higher in men than women in all glycaemic spectrums although women are at a higher relative risk for CVD compared to men –likely because women generally have a lower CVD risk profile, meaning T2D may have a proportionally greater impact on their overall risk [36]. Furthermore, CVD tends to develop later in women compared to men, primarily due to the protective effects of oestrogen on vascular health before menopause; after menopause, this hormonal protection is lost, contributing to a later onset of CVD in women [37,38].

In contrast to our findings, a systematic review and meta-analysis including 10 studies and more than 5 million participants found a higher excess risk for ESRD in women with T2D than in men (relative risk ratio = 1.38 (95% CI 1.22, 1.55; *p* = 0.114) [39].

Although sex differences in the risk for ESRD in patients with T2D remain unclear, some studies indicate that sex hormones play an important role in diabetic nephropathy, which is a common microvascular complication in patients with T2D and an important cause of ESRD in these patients. According to these studies [40,41], the hormone testosterone found in men accelerates the occurrence and progression of diabetic nephropathy, which may lead to ESRD. While oestrogen, the hormone found in women may have renoprotective effects. Additional studies are necessary to elucidate this relationship and understand the underlying mechanisms.

Women had higher average of health service use and long-term prescribing. It is established that gender is associated with differential patterns in healthcare-seeking. A study investigating the age-period-cohort effect in gender patterns of health service utilisation in Australia found that men engaged less in healthcare services compared to women of the same age and time period and that men’s pattern of health service use across time is likely explained by ageing [42]. On the other hand, a UK study [43] found that men had 32% fewer consultations than women, with primary care visits varying by age, with the biggest gender gap in those aged 16–60; however, the average number of consultation was similar between men and women who received medication for similar underlying comorbidities. Our findings suggest health-seeking behaviour among women compared to men, who, as a result of routine care, may have more opportunities for prevention, which likely explains the higher proportion of disease trajectories among men.

Our study showed that both women and men who had a combination of a CVD/ESRD and a mental health condition after the T2D onset also had the highest average of primary care consultations, hospitalisations and long-term prescribing. This likely indicates the healthcare burden of these comorbidities at a population scale. Furthermore, this finding reflects the National Health Service’s (NHS) highly standardised and incentivised treatment pathways for CVD/ESRD, which encourage regular healthcare attendance. In contrast, such structured pathways are less established for mental health conditions, suggesting that the presence of CVD/ESRD primarily drives the increased healthcare utilisation observed in this group. Individuals with T2D who have physical and mental comorbidities tend to have higher average of hospital admissions, longer hospital stays, and more emergency room visits, including unplanned ones [44,45]. Managing T2D becomes even more complicated when considering the impact of these comorbidities on treatment choices and healthcare services [46]. Therefore, it is crucial to adopt a comprehensive, patient-centred approach that addresses both physical and mental health needs to manage T2D effectively.

We found that hypertension was the most likely event after the onset of T2D for almost all age groups in both sexes, except for women in the youngest group. Our results support previous findings that T2D and hypertension are highly prevalent conditions that often coexist, with a possible bidirectional association between them [47–50] given that insulin resistance has a significant impact on the vasculature [51] and disturbances in carbohydrate metabolism are more common in hypertensive individuals [52].

We found that men had an earlier onset of health events after the diagnosis of T2D than women. Anxiety, depression, or somatoform disorders occurred at the youngest average age for both. The literature shows that women benefit from the protective effects of oestrogen, delaying the onset of cardiovascular conditions [53,54]. However, early menopause in women is linked to an increased risk of cardiovascular diseases [55], especially women with T2D, who show an even greater CVD risk, highlighting the importance of gender-specific health strategies. The earlier onset of mental health conditions could mean that most people will have developed these conditions before reaching older age [56]. The elderly often face different health challenges, such as chronic physical conditions, which can overshadow or mask the symptoms of a mental health condition [57]. Another striking finding is that women and men with mental health conditions following the onset of T2D had the earliest age at death, suggesting premature mortality in individuals with these comorbidities. Remarkably, men who had the trajectory T2D>ADS>DEATH died on average 9 years younger (61 years of age) than women in the same trajectory (70 years of age), although they had considerably lower average of long-term prescribing, consultation, and hospitalisation than women. This finding is supported by previous studies [30,58] showing that mental health conditions in individuals with T2D were associated with an increased mortality risk, which may be due to a combination of factors such as poor management of their physical health conditions, behavioural factors, and the direct effects of mental illness as well as, particularly in men, possible undertreatment and lower engagement with healthcare [59]. However, premature mortality in individuals with T2D and comorbid mental health conditions, such as depression, anxiety or somatoform disorder, remains under-investigated.

We found that individuals with mental health condition in their trajectories also had the highest average of healthcare service utilisation and long-term prescribing. Our results support previous studies where depression is associated with higher risks of complications and healthcare utilisation among T2D individuals [44,60,61]. Managing both T2D and mental health conditions requires a multidisciplinary approach that is likely to result in regular check-ups, frequent primary care consultations and long-term prescribing contributing to increased healthcare utilisation [44,60,61]. Also, a severe mental health condition can affect the individual’s ability to adhere to their diabetes management plans, including medication intake, behavioural changes and monitoring of blood sugar levels [62,63].

This study provides the first sex-stratified characterisation of disease trajectories following T2D diagnosis in a large, population-based UK cohort, revealing that the burden of T2D-related comorbidity is not evenly distributed between women and men. Women disproportionately accumulate mental health comorbidities earlier and face greater healthcare utilisation, while men experience earlier cardio-renal deterioration and, among those with mental health trajectories, markedly premature mortality. These findings indicate that knowing a complication is likely to occur is not enough; knowing when complications tend to occur and in what order may help translate population-level evidence into more targeted clinical action.

Crucially, the order and timing of conditions may have meaningful implications for how clinicians respond, even if these population-level patterns will not apply uniformly to every individual. For example, the evidence that people with T2D are at higher risk of both depression and heart disease describes two risks in isolation. However, the evidence that a mental health condition tends to emerge first in the average patient and that its presence may then accelerate the path toward cardiovascular disease and earlier death suggests there could be a window of opportunity for early intervention that might otherwise be missed. Clinicians and policymakers should make sure that routine mental health screening is integrated into T2D care pathways, while strengthening early cardio-renal surveillance and engagement strategies. These findings nonetheless require replication in other settings and populations, and future research incorporating causal methods will be important to determine whether intervening at these critical points in the disease trajectory can meaningfully alter outcomes.

### Strengths and limitations

The utilisation of a large-scale, nationally representative database and the application of a robust longitudinal analysis to elucidate disease acquisition after T2D onset is a major strength of our study. The multi-state analysis enabled us to understand the sequence and which combination of cardiovascular and renal diseases and mental health conditions are more common in women and men with T2D to support target interventions and personalised healthcare delivery across different age groups.

Our findings emphasise the need for healthcare strategies that address both physical and mental health aspects, particularly for women who exhibit a higher probability of having a mental health condition post-T2D onset. Likewise, early interventions are crucial for individuals with a combination of T2D and a mental health condition, who had premature mortality compared to individuals with a different combination of the conditions investigated. The detailed analysis of sociodemographic variables, health service utilisation, and long-term prescribing patterns contributes to the understanding of how these factors influence disease trajectories and health outcomes, providing a comprehensive view that can inform targeted interventions and policy decisions.

Our study findings should be interpreted considering the following limitations. First, our analysis did not specify mortality related to cardiovascular outcomes. Instead, all-cause mortality was considered. Second, hypertension is frequently diagnosed shortly after T2D due to the concurrent screening and management of these conditions. Consequently, it is likely that the higher proportion of the transition between T2D and hypertension underestimated the real transition between T2D and mental health or cardiovascular conditions and ESRD. An additional limitation is the sample size; our cohort was not large enough to analyse the trajectories according to ethnicity, especially minority ethnic groups, such as South Asian, Black African/Caribbean, Mixed, Chinese and other groups with fewer to no health state transitions in the observed 10-year period. Selection bias is also likely to occur –patients in EHR represent those who sought care, missing the healthier population or those with barriers to access.

Regarding the generalisability of our findings, the UK’s NHS provides a largely universal, free-at-point-of-care system with structured primary care as the main gateway to specialist services, and CPRD GOLD captures data from a nationally representative sample of GP practices in England. While these features support the internal validity and breadth of our findings, caution is warranted when extrapolating to countries with fragmented or insurance-based healthcare systems, where differential access to care may alter the observed trajectories, particularly for mental health conditions. Nevertheless, the sex-specific patterns we describe—including higher percentage of mental health diagnoses in women and higher cardiovascular burden in men—are broadly consistent with international evidence, suggesting that the underlying biological and social mechanisms may transcend the specificities of the UK healthcare context.

Even though our results show differences across sex in the diagnosis of a mental health condition, and despite the complex interplay behind them, it is not clear whether these differences could also be attributable to reporting bias among men or a secondary effect from a higher healthcare service utilisation among women.

Furthermore, using multi-state modelling in multimorbidity studies presents a few challenges. First, including more diseases in the analysis exponentially increases the complexity of the model outputs. Second, the conditions included must be meticulously operationalised to avoid over-aggregation or over-granularity.

Finally, given the descriptive nature of our study, we did not incorporate biomarkers or clinical measurements (e.g., HbA1c, blood pressure, eGFR), nor did we account for treatment exposures such as glucose-lowering, cardiovascular, or psychotropic medications and the focus on transition probabilities without explicit quantification of absolute and relative risks may limit direct clinical interpretability. Nevertheless, by mapping the sequence and combination of complications across sex and age groups, this study provides a foundational trajectory framework that future research can extend by incorporating biomarker data, medication exposures, and causal inference methods to translate these sex-specific patterns into targeted, personalised prevention and treatment strategies.

## Supporting information

S1 Protocol Study protocol for research using the Clinical Practice Research Datalink (CPRD). (PDF)

S1 Checklist RECORD Statement. Checklist of items, extended from the STROBE statement, that should be reported in observational studies using routinely collected health data. Adapted from Benchimol EI, Smeeth L, Guttmann A, Harron K, Moher D, Petersen I, and colleagues. The REporting of studies Conducted using Observational Routinely-collected health Data (RECORD) Statement. PLoS Med. 2015;12(10):e1001885. https://doi.org/10.1371/journal.pmed.1001885. Available under a Creative Commons Attribution 4.0 International License (CC BY 4.0): https://creativecommons.org/licenses/by/4.0/.
(PDF)

S1 Table Age distribution of the T2D cohort according to trajectories following the onset of type 2 diabetes. The proportions are related to the entire T2D cohort (*n* = 28,720). Data are *n* (%) or mean (SD).
(PDF)

S2 Table Sociodemographic characteristics and health service utilisation of the T2D cohort according to trajectories following the onset of type 2 diabetes. The proportions are related to the entire T2D cohort (*n* = 28,720).
(PDF)

S3 Table Probabilities of having experienced each competing next state following the onset of T2D and average time to the next state for women between 16 and 39 years old. (PDF)

S4 Table Probabilities of having experienced each competing next state following the onset of T2D and average time to the next state for women between 40 and 59 years old. (PDF)

S5 Table Probabilities of having experienced each competing next state following the onset of T2D and average time to the next state for women 60 years and older. (PDF)

S6 Table Probabilities of having experienced each competing next state following the onset of T2D and average time to the next state for men between 16 and 39 years old. (PDF)

S7 Table Probabilities of having experienced each competing next state following the onset of T2D and average time to the next state for men between 40 and 59 years old. (PDF)

S8 Table Probabilities of having experienced each competing next state following the onset of T2D and average time to the next state for men 60 years and older. (PDF)

S9 Table Total frequency and proportion of each health event by sex. (PDF)

S1 Fig Multi-state diagram for the entire T2D cohort. **Number and proportion of individuals who had each competing next state.** The boxes represent the 11 possible states, and the arrows represent the 19 transitions. ADS = anxiety, depression or somatoform disorder. CVD = atrial fibrillation and flutter, cerebrovascular disease, coronary heart disease, heart failure or peripheral arterial disease. ESRD = end-stage renal disease. HPT = hypertension. T2D = type 2 diabetes.
(TIF)

S2 Fig Average probabilities of each competing second state following the onset of T2D by sex and age groups. Points represent the estimated average probability of the event, and horizontal bars represent the corresponding 95% confidence intervals. ADS = anxiety, depression or somatoform disorder. CVD = atrial fibrillation and flutter, cerebrovascular disease, coronary heart disease, heart failure or peripheral arterial disease. ESRD = end-stage renal disease. HPTN = hypertension. T2D = type 2 diabetes.
(TIF)

S3 Fig Average probabilities of each competing third state following the second state by sex and age groups. Points represent the estimated average probability of the event, and horizontal bars represent the corresponding 95% confidence intervals. ADS = anxiety, depression or somatoform disorder. CVD = atrial fibrillation and flutter, cerebrovascular disease, coronary heart disease, heart failure or peripheral arterial disease. ESRD = end-stage renal disease. HPTN = hypertension. T2D = type 2 diabetes.
(TIF)

S4 Fig Average time to each competing second state following the onset of T2D by sex and age groups. Points represent the estimated average probability of the event, and horizontal bars represent the corresponding 95% confidence intervals. ADS = anxiety, depression or somatoform disorder. CVD = atrial fibrillation and flutter, cerebrovascular disease, coronary heart disease, heart failure or peripheral arterial disease. ESRD = end-stage renal disease. HPTN = hypertension. T2D = type 2 diabetes.
(TIF)

S5 Fig Average time to each competing third state following the second state by sex and age groups. Points represent the estimated average probability of the event, and horizontal bars represent the corresponding 95% confidence intervals. ADS = anxiety, depression or somatoform disorder. CVD = atrial fibrillation and flutter, cerebrovascular disease, coronary heart disease, heart failure or peripheral arterial disease. ESRD = end-stage renal disease. HPTN = hypertension. T2D = type 2 diabetes.
(TIF)

S6 Fig Average time to death after each competing third state following the onset of T2D by sex and age groups. Points represent the estimated average probability of the event, and horizontal bars represent the corresponding 95% confidence intervals. ADS = anxiety, depression or somatoform disorder. CVD = atrial fibrillation and flutter, cerebrovascular disease, coronary heart disease, heart failure or peripheral arterial disease. ESRD = end-stage renal disease. HPTN = hypertension. T2D = type 2 diabetes.
(TIF)

S7 Fig T2D cohort’s median age entering each health state. ADS = anxiety, depression or somatoform disorder. CVD = atrial fibrillation and flutter, cerebrovascular disease, coronary heart disease, heart failure or peripheral arterial disease. ESRD = end-stage renal disease. HTN = hypertension. T2D = type 2 diabetes.
(TIF)

## Abbreviations

BNF: British National Formulary
CPRD: Clinical Practice Research Datalink
EHR: electronic health record
ESRD: end-stage renal disease
GDPR: General Data Protection Regulation
GP: general practice
IMD: Index of Multiple Deprivation
ONS: Office for National Statistics.
