# Supplementary material for: Sex differences in trajectories to cardio-renal and mental health outcomes following the onset of type 2 diabetes: An observational cohort study using multi-state analysis in the UK Clinical Practice Research Datalink
Source: PLoS Med. 2026 Aug 25;23(8):e1005196. doi: 10.1371/journal.pmed.1005196 (PMC13505941; doi:10.1371/journal.pmed.1005196)
Supplement: Supplementary file 3 [file pmed.1005196.s003.pdf]

S1 Table. Age distribution of the T2D cohort according to trajectories following the onset of type 2 diabetes. The proportions are related to the entire T2D cohort (n= 28,720).

	n (%)	Sex		Age at onset of T2D	Age group at T2D onset			Age at death
		Female	Male		16-39	40-59	60+	
**T2D cohort**	28,720 (100)	10,903 (38)	17,817 (62)	55 (14)	3,923 (14)	14,314 (50)	10,483 (36)	73 (14)
**Individuals who had an event after the T2D onset**	11,161 (39)	4,184 (37)	6,977 (63)	59 (14)	1,017 (9)	4,871 (44)	5,273 (47)	72 (14)
**>HTN**	5,953 (20.7)	2,202 (37)	3,751 (63)	57 (13)	456 (8)	2869 (48)	2628 (44)	-
HTN>ADS	374 (6.3)	189 (51)	185 (49)	54 (13)	41 (11)	206 (55)	127 (34)	-
HTN>ADS>DEATH	46 (12.3)	33 (72)	13 (28)	68 (14)	[redacted]	10 (26)	35 (72)	73 (13)
HTN>CVD/ESRD	745 (12.5)	234 (31)	511 (69)	63 (13)	32 (4)	263 (35)	450 (60)	-
HTN>CVD/ESRD>DEATH	184 (24.7)	66 (36)	118 (64)	70 (12)	[redacted]	35 (19)	145 (80)	76 (12)
HTN>DEATH	367 (6.2)	141 (38)	226 (62)	68 (13)	[redacted]	85 (23)	275 (75)	73 (13)
**>ADS**	1,835 (6.4)	882 (48)	953 (52)	49 (14)	439 (24)	1003 (55)	393 (21)	-
ADS>HTN	313 (17.1)	145 (46)	168 (54)	52 (13)	51 (16)	185 (59)	77 (25)	-
ADS>HTN>DEATH	28 (8.9)	18 (64)	10 (36)	67 (14)	0 (0)	9 (32)	19 (68)	73 (14)
ADS>CVD/ESRD	133 (7.2)	54 (41)	79 (59)	57 (14)	13 (10)	65 (49)	55 (41)	-
ADS>CVD/ESRD>DEATH	32 (24.1)	12 (38)	20 (62)	66 (14)	[redacted]	10 (25)	25 (72)	71 (14)
ADS>DEATH	123 (6.7)	68 (55)	55 (45)	63 (15)	[redacted]	45 (37)	70 (58)	67 (15)
**>CVD/ESRD**	2,079 (7.2)	582 (28)	1,497 (72)	64 (13)	84 (4)	723 (35)	1272 (61)	-
CVD/ESRD>HTN	576 (27.7)	134 (23)	442 (77)	63 (12)	16 (3)	210 (36)	350 (61)	-
CVD/ESRD>HTN>DEATH	132 (22.9)	37 (28)	95 (72)	72 (11)	[redacted]	20 (14)	115 (85)	77 (11)
CVD/ESRD>ADS	103 (5.0)	38 (37)	65 (63)	61 (15)	[redacted]	45 (44)	55 (51)	-
CVD/ESRD>ADS>DEATH	23 (22.3)	12 (52)	11 (48)	70 (16)	[redacted]	[redacted]	20 (78)	75 (16)
CVD/ESRD>DEATH	421 (20.3)	150 (36)	271 (64)	72 (12)	[redacted]	60 (14)	357 (85)	76 (12)
**DEATH**	1,294 (4.5)	518 (40)	776 (60)	69 (14)	38 (3)	276 (21)	980 (76)	71 (14)

Data are n (%) or mean (SD).

The proportions are related to the entire T2D cohort (n= 28,720).
Any counts describing ≤ 7 patients were redacted to avoid primary disclosure, and the complementary values that could be combined with other results to reveal information about individuals or groups were rounded to the nearest five. This is the reason that for some categorical variables, the counts will not sum up to the total column. The proportions related to the rounded values will sum up to 100%.
ADS = anxiety, depression or somatoform disorder. CVD = atrial fibrillation and flutter, cerebrovascular disease, coronary heart disease, heart failure or peripheral arterial disease. ESRD = end-stage renal disease. HTN = hypertension. T2D = type 2 diabetes.

	n (%)	IMD (quintiles)					Long-term prescribing[1]	Consultation	Hospitalisation
		1Q (least deprived)	2Q	3Q	4Q	5Q (greatest deprived)			
**T2D cohort**	28,720 (100)	5,413 (19)	5,485 (19)	5,967 (21)	6,155 (21)	5,700 (20)	2 (4)	55 (107)	2 (7)
**Individuals who had an event after the T2D onset**	11,161 (39)	2,065 (19)	2,132 (19)	2,320 (21)	2,439 (22)	2,205 (20)	3 (5)	74 (128)	3 (10)
**>HTN**	5,953 (20.7)	1133 (19)	1129 (19)	1230 (21)	1308 (22)	1153 (19)	3 (5)	75 (128)	2 (7)
HTN>ADS	374 (6.3)	63 (17)	48 (13)	86 (23)	79 (21)	98 (26)	6 (7)	122 (170)	4 (9)
HTN>ADS>DEATH	46 (12.3)	[redacted]	[redacted]	10 (24)	15 (28)	10 (20)	6 (7)	110 (142)	7 (10)
HTN>CVD/ESRD	745 (12.5)	132 (18)	161 (22)	148 (20)	165 (22)	139 (19)	5 (7)	114 (160)	5 (10)
HTN>CVD/ESRD>DEATH	184 (24.7)	30 (16)	45 (24)	41 (22)	33 (18)	35 (19)	6 (8)	123 (175)	7 (12)
HTN>DEATH	367 (6.2)	72 (20)	85 (23)	80 (22)	69 (19)	61 (17)	3 (5)	67 (110)	5 (12)
**>ADS**	1,835 (6.4)	276 (15)	318 (17)	383 (21)	424 (23)	434 (24)	4 (6)	91 (139)	4 (19)
ADS>HTN	313 (17.1)	49 (16)	43 (14)	69 (22)	82 (26)	70 (22)	6 (7)	126 (164)	6 (41)
ADS>HTN>DEATH	28 (8.9)	[redacted]	[redacted]	10 (25)	10 (29)	[redacted]	6 (8)	137 (165)	10 (14)
ADS>CVD/ESRD	133 (7.2)	22 (17)	22 (17)	33 (25)	25 (19)	31 (23)	6 (8)	136 (165)	6 (12)
ADS>CVD/ESRD>DEATH	32 (24.1)	[redacted]	10 (28)	[redacted]	[redacted]	[redacted]	5 (9)	101 (177)	4 (6)
ADS>DEATH	123 (6.7)	15 (12)	32 (26)	24 (20)	25 (20)	27 (22)	4 (6)	80 (121)	6 (18)
**>CVD/ESRD**	2,079 (7.2)	384 (18)	426 (20)	440 (21)	432 (21)	397 (19)	3 (6)	78 (137)	3 (8)
CVD/ESRD>HTN	576 (27.7)	117 (20)	109 (19)	130 (23)	109 (19)	111 (19)	4 (6)	97 (148)	4 (8)
CVD/ESRD>HTN>DEATH	132 (22.9)	23 (17)	27 (20)	33 (25)	26 (20)	23 (17)	5 (7)	97 (144)	6 (10)
CVD/ESRD>ADS	103 (5.0)	15 (15)	28 (27)	15 (15)	23 (22)	22 (21)	6 (8)	130 (185)	6 (9)
CVD/ESRD>ADS>DEATH	23 (22.3)	[redacted]	[redacted]	[redacted]	[redacted]	[redacted]	7 (9)	131 (181)	9 (11)
CVD/ESRD>DEATH	421 (20.3)	64 (15)	92 (22)	92 (22)	84 (20)	89 (21)	3 (6)	69 (126)	4 (11)
**DEATH**	1,294 (4.5)	272 (21)	259 (20)	267 (21)	275 (21)	221 (17)	2 (4)	42 (88)	3 (10)

Data are n (%) or mean (SD).

The proportions are related to the entire T2D cohort (n= 28,720).

[1] Counts of unique BNF subparagraphs from which an individual had continuous prescribing (3 or more prescriptions that occur in a year).
Any counts describing ≤ 7 patients were redacted to avoid primary disclosure, and the complimentary values that could be combined with other results to reveal information about individuals or groups were rounded to the nearest five. This is the reason that for some categorical variables, the counts sum up will not match the total column. The proportions related to the rounded values will sum up to 100%.
ADS = anxiety, depression or somatoform disorder. BNF = British National Formulary. CVD = atrial fibrillation and flutter, cerebrovascular disease, coronary heart disease, heart failure or peripheral arterial disease. ESRD = end-stage renal disease. HTN = hypertension. IMD = Index of Multiple Deprivation. Q = quintile. T2D = type 2 diabetes.
