# Supplementary material for: Sex differences in trajectories to cardio-renal and mental health outcomes following the onset of type 2 diabetes: An observational cohort study using multi-state analysis in the UK Clinical Practice Research Datalink
Source: PLoS Med. 2026 Aug 25;23(8):e1005196. doi: 10.1371/journal.pmed.1005196 (PMC13505941; doi:10.1371/journal.pmed.1005196)
Supplement: Supplementary file 6 [file pmed.1005196.s006.pdf]

S4 Table. Probabilities of having experienced each competing next state following the onset of T2D and average time to the next state for women between 40 and 59 years old.

	**40 to 59 years old (n= 4,893)**			
**Trajectory after the onset of T2D**	**n (%)**	**No event (%)**	**Probability of the next event (competing risk)**	**Average time (years) to the next event**
**T2D**	**4,893 (100)**	**3,286 (67,2)**	**-**	**-**
**>HTN**	956 (19.5)	763 (79.8)	0.54 (0.53, 0.55)	3.38 (3.32, 3.48)
HTN>ADS	101 (10.6)	312 (73.1)	0.5 (0.42, 0.59)	3.93 (3.73, 4.19)
HTN>ADS>DEATH	20 (4.7)	-	-	3.44 (2.99, 4.13)
HTN>CVD/ESRD	64 (6.7)	81 (55.9)	0.39 (0.31, 0.47)	4.08 (3.85, 4.19)
HTN>CVD/ESRD>DEATH	10 (15.6)	-	-	3.26 (2.95, 3.48)
HTN>DEATH	28 (2.9)	-	0.11 (0.09, 0.13)	3.54 (3.29, 3.82)
**>ADS**	427 (8.7)	312 (73.1)	0.28 (0.27, 0.3)	3.48 (3.4, 3.69)
ADS>HTN	77 (18.0)	69 (89.6)	0.66 (0.56, 0.74)	3.86 (3.66, 4.16)
ADS>HTN>DEATH	10 (8.9)	-	-	3.97 (3.06, 4.6)
ADS>CVD/ESRD	18 (4.2)	16 (88.9)	0.21 (0.14, 0.27)	3.95 (3.68, 4.37)
ADS>CVD/ESRD>DEATH	[redacted]	-	-	2.82 (2.29, 3.4)
ADS>DEATH	20 (4.7)	-	0.14 (0.1, 0.18)	3.25 (2.82, 3.7)
**>CVD/ESRD**	145 (3.0)	81 (55.9)	0.12 (0.11, 0.13)	3.75 (3.67, 3.98)
CVD/ESRD>HTN	38 (26.2)	34 (89.5)	0.58 (0.44, 0.67)	3.08 (2.82, 3.26)
CVD/ESRD>HTN>DEATH	[redacted]	-	-	3.86 (3.43, 4.14)
CVD/ESRD>ADS	12 (8.3)	10 (83.3)	0.23 (0.17, 0.38)	3.26 (2.83, 3.6)
CVD/ESRD>ADS>DEATH	[redacted]	-	-	4.32 (3.41, 5.2)
CVD/ESRD>DEATH	14 (9.7)	-	0.18 (0.16, 0.2)	2.34 (2.09, 2.6)
**>DEATH**	79 (1.6)	-	0.05 (0.05, 0.06)	3.42 (3.1, 3.56)

Any counts describing ≤ 7 patients were redacted to avoid primary disclosure, and the complementary values that could be combined with other results to reveal information about individuals or groups were rounded to the nearest five. This is the reason that for some categorical variables, the counts will not sum up to the total column. The proportions related to the rounded values will sum up to 100%.

ADS = anxiety, depression or somatoform disorder. CVD = atrial fibrillation and flutter, cerebrovascular disease, coronary heart disease, heart failure or peripheral arterial disease. ESRD = end-stage renal disease. HTN = hypertension. T2D = type 2 diabetes.
