# Supplementary material for: Sex differences in trajectories to cardio-renal and mental health outcomes following the onset of type 2 diabetes: An observational cohort study using multi-state analysis in the UK Clinical Practice Research Datalink
Source: PLoS Med. 2026 Aug 25;23(8):e1005196. doi: 10.1371/journal.pmed.1005196 (PMC13505941; doi:10.1371/journal.pmed.1005196)
Supplement: Supplementary file 7 [file pmed.1005196.s007.pdf]

S5 Table. Probabilities of having experienced each competing next state following the onset of T2D and average time to the next state for women 60 years and older.

	60 years and older (n= 4,354)			
**Trajectory after the onset of T2D**	**n (%)**	**No event (%)**	**Probability of the next event (competing risk)**	**Average time (years) to the next event**
**T2D**	**4,354 (100)**	**2,212 (50.8)**	**-**	**-**
**>HTN**	1,096 (25.2)	749 (68.3)	0.5 (0.48, 0.51)	3.05 (3.05, 3.2)
HTN>ADS	71 (6.5)	47 (66.2)	0.22 (0.18, 0.26)	3.58 (3.5, 3.99)
HTN>ADS>DEATH	47 (21.9)	-	-	3.11 (2.65, 3.72)
HTN>CVD/ESRD	165 (15.1)	110 (66.7)	0.5 (0.44, 0.54)	3.78 (3.66, 4.03)
HTN>CVD/ESRD>DEATH	55 (33.3)	-	-	2.87 (2.61, 3.13)
HTN>DEATH	111 (10.1)	-	0.28 (0.24, 0.31)	3.32 (3.19, 3.61)
**>ADS**	215 (4.9)	97 (45.1)	0.11 (0.09, 0.11)	3.28 (3.14, 3.44)
ADS>HTN	42 (19.5)	32 (76.2)	0.4 (0.32, 0.46)	3.44 (3.18, 3.64)
ADS>HTN>DEATH	10 (23.8)	-	-	3.42 (2.66, 4.14)
ADS>CVD/ESRD	29 (13.5)	19 (65.5)	0.26 (0.22, 0.32)	3.48 (3.15, 3.88)
ADS>CVD/ESRD>DEATH	10 (34.5)	-	-	2.41 (1.95, 2.96)
ADS>DEATH	47 (21.9)	-	0.34 (0.27, 0.41)	2.75 (2.39, 3.13)
**>CVD/ESRD**	410 (9.4)	160 (39.0)	0.21 (0.21, 0.23)	3.58 (3.36, 3.69)
CVD/ESRD>HTN	91 (22.2)	59 (64.8)	0.41 (0.36, 0.45)	2.62 (2.44, 2.86)
CVD/ESRD>HTN>DEATH	32 (35.2)	-	-	3.27 (2.98, 3.62)
CVD/ESRD>ADS	24 (5.9)	14 (58.3)	0.11 (0.08, 0.15)	2.82 (2.35, 3.28)
CVD/ESRD>ADS>DEATH	10 (41.7)	-	-	3.54 (2.65, 4.42)
CVD/ESRD>DEATH	135 (32.9)	-	0.48 (0.43, 0.52)	1.96 (1.76, 2.18)
**>DEATH**	421 (9.7)	-	0.19 (0.17, 0.2)	3.04 (2.93, 3.23)

Any counts describing ≤ 7 patients were redacted to avoid primary disclosure, and the complementary values that could be combined with other results to reveal information about individuals or groups were rounded to the nearest five. This is the reason that for some categorical variables, the counts will not sum up to the total column. The proportions related to the rounded values will sum up to 100%.

ADS = anxiety, depression or somatoform disorder. CVD = atrial fibrillation and flutter, cerebrovascular disease, coronary heart disease, heart failure or peripheral arterial disease. ESRD = end-stage renal disease. HTN = hypertension. T2D = type 2 diabetes.
