# Supplementary material for: Sex differences in trajectories to cardio-renal and mental health outcomes following the onset of type 2 diabetes: An observational cohort study using multi-state analysis in the UK Clinical Practice Research Datalink
Source: PLoS Med. 2026 Aug 25;23(8):e1005196. doi: 10.1371/journal.pmed.1005196 (PMC13505941; doi:10.1371/journal.pmed.1005196)
Supplement: Supplementary file 8 [file pmed.1005196.s008.pdf]

S6 Table. Probabilities of having experienced each competing next state following the onset of T2D and average time to the next state for men between 16 and 39 years old.

	**16 to 39 years old (n= 2,267)**			
**Trajectory after the onset of T2D**	**n (%)**	**No event (%)**	**Probability of the next event**	**Average time to the next event**
**T2D**	**2,267 (100)**	**1,685 (74.3)**	**-**	**-**
**>HTN**	306 (13.5)	252 (82.4)	0.45 (0.43, 0.46)	3.6 (3.48, 3.69)
HTN>ADS	25 (7.8)	25 (95.8)	0.43 (0.33, 0.5)	3.92 (3.73, 4.22)
HTN>ADS>DEATH	[redacted]	-	-	3.74 (2.99, 4.29)
HTN>CVD/ESRD	25 (8.8)	25 (96.3)	0.52 (0.45, 0.59)	4.13 (3.92, 4.3)
HTN>CVD/ESRD>DEATH	[redacted]	-	-	3.36 (2.98, 3.61)
HTN>DEATH	[redacted]	-	0.05 (0.03, 0.1)	3.55 (3.27, 3.97)
**>ADS**	199 (8.8)	163 (81.9)	0.38 (0.37, 0.42)	3.74 (3.6, 3.86)
ADS>HTN	25 (12.6)	25 (100)	0.73 (0.56, 0.81)	4.08 (3.86, 4.31)
ADS>HTN>DEATH	0	-	-	-
ADS>CVD/ESRD	[redacted]	[redacted]	0.23 (0.15, 0.37)	4.24 (3.85, 4.53)
ADS>CVD/ESRD>DEATH	[redacted]	-	-	2.82 (1.89, 3.36)
ADS>DEATH	[redacted]	-	0.05 (0.03, 0.13)	3.38 (3.1, 3.98)
**>CVD/ESRD**	57 (2.5)	40 (70.2)	0.13 (0.1, 0.14)	4.03 (3.79, 4.21)
CVD/ESRD>HTN	10 (19.3)	10 (100)	0.71 (0.52, 0.74)	3.33 (3.02, 3.58)
CVD/ESRD>HTN>DEATH	0	-	-	3.99 (3.4, 4.17)
CVD/ESRD>ADS	[redacted]	[redacted]	0.19 (0.14, 0.36)	3.58 (2.92, 3.96)
CVD/ESRD>ADS>DEATH	[redacted]	-	-	4.32 (2.68, 5.26)
CVD/ESRD>DEATH	[redacted]	-	0.1 (0.05, 0.19)	2.74 (2.28, 2.91)
**>DEATH**	20 (0.9)	-	0.04 (0.03, 0.05)	3.99 (3.2, 3.94)

Any counts describing $\leq$ 7 patients were redacted to avoid primary disclosure, and the complimentary values that could be combined with other results to reveal information about individuals or groups were rounded to the nearest five. This is the reason that for some categorical variables, the counts sum up will not match the total column. The proportions related to the rounded values will sum up to 100%.

ADS = anxiety, depression or somatoform disorder. CVD = atrial fibrillation and flutter, cerebrovascular disease, coronary heart disease, heart failure or peripheral arterial disease. ESRD = end-stage renal disease. HTN = hypertension. T2D = type 2 diabetes.
