# Supplementary material for: Sex differences in trajectories to cardio-renal and mental health outcomes following the onset of type 2 diabetes: An observational cohort study using multi-state analysis in the UK Clinical Practice Research Datalink
Source: PLoS Med. 2026 Aug 25;23(8):e1005196. doi: 10.1371/journal.pmed.1005196 (PMC13505941; doi:10.1371/journal.pmed.1005196)
Supplement: Supplementary file 11 [file pmed.1005196.s011.pdf]

S9 Table. Total frequency and proportion of each health event by sex.

	Women	Men
**Health event**	**N (%)**	**N (%)**
T2D	10,903 (100)	17,817 (100)
HTN	2,481 (22.8%)	4,361 (24.5%)
ADS	1,109 (10.2%)	1,203 (6.8%)
CVD/ESRD	870 (8.0%)	2,087 (11.7%)
DEATH	1,055 (9.7%)	1,595 (9.0%)

ADS = anxiety, depression or somatoform disorder. CVD = atrial fibrillation and flutter, cerebrovascular disease, coronary heart disease, heart failure or peripheral arterial disease. ESRD = end-stage renal disease. HTN = hypertension. T2D = type 2 diabetes.
